# Anisotropic Gold Nanoconjugates Bearing Immunoinformatically Designed T-Cell Epitope-Containing Peptides: Comparative Evaluation in THP-1 and Human Primary Macrophages

**DOI:** 10.3390/ijms27188400

**Published:** 2026-09-21

**Authors:** Braulio Contreras-Trigo, Enrique Guzmán-Gutiérrez, Eduardo Zúñiga, Fabián Sáez-Ahumada, Víctor Fica-León, Simón Guerrero, Dariela Núñez, Andrea Sánchez, Carmen Molina-Paris, Bostjan Kobe, Víctor Díaz-García, Patricio Oyarzún

**Affiliations:** 1 Facultad de Ingeniería, Universidad San Sebastián, Concepción 4081339, Chile; bcontrerast@docente.uss.cl (B.C.-T.); vfical@correo.uss.cl (V.F.-L.); victor.diazg@uss.cl (V.D.-G.); 2 Departamento de Bioquímica Clínica e Inmunología, Facultad de Farmacia, Universidad de Concepción, Concepción 4070386, Chile; fabsaez@udec.cl (F.S.-A.); andrsac@udec.cl (A.S.); 3 Facultad de Ciencias Químicas y Farmacéuticas, Universidad de Chile, Santiago 380492, Chile; ezunigaz@docente.uss.cl (E.Z.); simon.guerrero@ciq.uchile.cl (S.G.); 4 Departamento de Química Ambiental, Facultad de Ciencias, Universidad Católica de la Santísima Concepción, Concepción 4030000, Chile; dnunez@ucsc.cl; 5 Centro de Investigación en Biodiversidad y Ambientes Sustentables (CIBAS), Universidad Católica de la Santísima Concepción, Concepción 4030000, Chile; 6 Theoretical Biology and Biophysics Group, Los Alamos National Laboratory, Los Alamos, NM 87545, USA; molina-paris@lanl.gov; 7 School of Chemistry and Molecular Biosciences, Institute for Molecular Bioscience, Australian Infectious Diseases Research Centre, The University of Queensland, Brisbane, QLD 4072, Australia; b.kobe@uq.edu.au

**Keywords:** gold nanoparticles, nanorods, nanostars, peptide nanovaccine, T-cell epitope, macrophages, THP-1, primary human macrophages, cytotoxicity, antigen processing

## Abstract

Gold nanoparticles (AuNPs) are promising peptide carriers whose size and anisotropic morphology may influence their interactions with antigen-presenting cells. However, current knowledge of the morphology-dependent effects of AuNPs and the role of peptide design in human macrophage responses remains incomplete. Furthermore, most studies have been performed in THP-1-derived macrophages as the main in vitro macrophage-like model, despite human primary macrophages (HPMs) providing a more physiologically representative system. Anisotropic AuNPs with two morphologies, nanorods (AuNRs) and nanostars (AuNSs), each synthesized in two sizes, were characterized and conjugated to three influenza M2 T-cell-epitope constructs containing CD4+ (P1) and CD8+ T-cell epitopes (P2), individually, and both combined in a single multiepitope construct (P3), optimized for intracellular processing using a novel bioinformatics tool coupled to Predivac 3.0. THP-1-derived macrophages and HPMs were exposed to these nanoconjugates at a fixed particle number concentration of 10 pM for 24 h and assessed for mitochondrial activity, cell death, reactive oxygen species, endolysosomal acidification, proteasome activity, and cathepsin B activity. The identity of the nanoparticle carrier, defined by its geometry together with its specific surface chemistry, rather than the type of conjugated peptide, dominated the cellular response. AuNRs, particularly long rods, reduced mitochondrial activity, increased cell death, and enhanced bulk 20S proteasome activity in both models, even though at equal particle number they delivered 21- to 53-fold less gold mass and 6- to 12-fold less geometric surface area than AuNSs. The stimulatory effect induced by AuNRs-P1 and AuNRs-P2 on the proteasomal system may be associated with their specific peptide design features. By contrast, AuNSs preserved viability, maintained low oxidative stress, and produced strong acidification signals consistent with efficient trafficking to endolysosomal compartments; P2 and P3 conjugation further improved cytocompatibility and increased cathepsin B activity. HPMs were consistently more tolerant than THP-1 macrophages. The morphology-surface chemistry identity of the AuNP carrier is a key determinant of macrophage compatibility and of the relative engagement of the two degradative compartments assayed here. Functionalized AuNSs, especially P2- and P3-nanoconjugates, showed the best balance among biocompatibility, endolysosomal trafficking, and cathepsin B engagement. The proposed correspondence with MHC class I versus class II antigen-processing routes is a mechanistically informed hypothesis that remains to be further validated experimentally.

## 1. Introduction

Emerging infectious diseases (EIDs) are defined as infections whose incidence or geographic distribution is rapidly increasing or is expected to increase in the near future. Over the past decades, EIDs have emerged at an unprecedented rate due to multiple factors associated with globalization, environmental change, and increased human–animal interactions, posing serious threats to public health and global economies [1]. The COVID-19 pandemic caused by SARS-CoV-2 exemplifies the profound societal and economic consequences of newly emerging viral pathogens [2].

Traditional vaccine platforms based on live-attenuated or inactivated viruses are often associated with biosafety concerns and complex manufacturing requirements, making their development time-consuming, costly, and difficult to rapidly deploy during emerging pandemics. Moreover, these approaches do not always elicit strong cellular immune responses, which are essential for protection against many viral infections [3]. Consequently, there is growing interest in next-generation vaccine platforms capable of inducing robust and targeted immune responses while enabling faster development timelines [4]. One promising strategy is the development of epitope-based vaccines, which focus on short peptide sequences corresponding to antigenic determinants recognized by T cells (T-cell epitopes) [5]. These peptides are presented by major histocompatibility complex (MHC) molecules to T lymphocytes, initiating antigen-specific immune responses. In humans, MHC molecules, referred to as human leukocyte antigens (HLAs), are highly polymorphic proteins encoded by one of the most genetically diverse regions of the human genome [6]. Multi-epitope peptide vaccines are particularly attractive, as they offer the prospect for a more prominent role of HLA-restricted immune responses capable of inducing a broad range of immune specificities and targeting multiple viral antigens simultaneously [7]. A large body of evidence highlights CD8+ cytotoxic T-cells as key mediators of immunity against intracellular infections, while CD4+ T cells provide essential help for the generation of effective cellular responses and neutralizing antibodies [8,9].

However, peptide vaccines suffer from low intrinsic immunogenicity, as short peptide antigens are rapidly degraded in biological environments and often fail to efficiently stimulate antigen-presenting cells (APCs). Therefore, effective delivery systems or adjuvants are required to enhance antigen stability, promote cellular uptake, and facilitate antigen presentation through MHC pathways [10]. In this context, nanotechnology has emerged as a powerful platform for vaccine delivery and immune modulation. Nanomaterial-based carriers benefit from size-dependent interactions with immune cells, as their particulate nature enhances sensing and uptake by APCs compared with soluble antigens and increases persistence at the administration site [11]. In particular, gold nanoparticles (AuNPs) have attracted considerable attention due to their excellent biocompatibility, chemical stability, tunable size and shape, and ease of surface functionalization [12,13]. AuNPs can serve as scaffolds for multivalent antigen display, mimicking the repetitive antigenic patterns found on viral surfaces and thereby enhancing immune recognition, even in the absence of conventional adjuvants [14].

Cellular uptake of AuNPs is strongly size-dependent in the 10–100 nm range, with nanoparticles (NPs) of approximately 50 nm exhibiting the highest uptake efficiency [15]. While most nanovaccine studies have focused on spherical NPs, increasing evidence indicates that NP shape plays a critical role in determining biological responses. Structural parameters such as size, aspect ratio, shape anisotropy, and local surface curvature critically influence NP uptake, endocytic pathway selection, intracellular trafficking, endosomal processing, and downstream immune activation. These features regulate membrane wrapping, the energetic cost of internalization, biological corona formation, and recognition by phagocytic or antigen-presenting cells [16,17]. In gold nanomaterials, these effects are particularly relevant, as comparative studies have shown that nanorods, nanostars, and triangular particles display shape-dependent uptake and intracellular distribution profiles, while high-curvature or high-aspect-ratio structures may further modulate oxidative stress and macrophage-associated inflammatory responses.

Therefore, understanding these mechanisms is critical for improving delivery and targeting strategies in vaccinology and nanomedicine [18]. These effects are of particular interest for innate immune cells such as macrophages, which serve as key APCs in vaccine responses. The human leukemia monocytic cell line THP-1 is widely used as an in vitro model for macrophage-like cells and provides a standardized and reproducible model for evaluating NP–immune cell interactions, cytotoxicity, oxidative stress responses, and immunomodulatory effects. However, human primary macrophages (HPMs) provide a more physiologically representative system than transformed cell models, as they better capture donor-dependent variability, macrophage heterogeneity, and phenotype-specific responses. Indeed, the literature strongly supports the notion that THP-1-derived macrophages and HPMs are not equivalent models for studying NP-cell interactions. For example, primary human macrophages internalize functionalized polystyrene NPs (∼100 nm in diameter) more efficiently than PMA-differentiated monocytic THP-1 cells and through distinct mechanisms. Whereas primary macrophages predominantly rely on CD64-mediated phagocytosis, PMA-differentiated THP-1 cells mainly use dynamin II-dependent endocytosis or macropinocytosis [19]. In HPMs, the uptake of gold nanorods (diameters between 15 and 50 nm) has been shown to depend strongly on particle shape and surface chemistry, with nanorods being internalized more efficiently than nanospheres. Moreover, surface modifications can alter both the rate of internalization and the resulting inflammatory profile [20]. Likewise, in HPMs, gold nanorods are predominantly internalized through micropinocytosis as a dominant uptake pathway. By contrast, studies in immortalized cell lines have shown that multiple endocytic mechanisms, including clathrin-mediated endocytosis, caveolae-dependent pathways, and non-specific uptake, can operate simultaneously [21].

Despite growing interest in peptide-based AuNP platforms, side-by-side comparisons evaluating how peptide functionalization and NP morphology influence cytotoxicity and cellular responses in THP-1 cells versus HPMs remain limited. This gap in the literature provides a strong rationale for investigating whether these two cell models differ in their responses to peptide nanoconjugates under identical experimental conditions. Accordingly, in this study, we evaluate the cytotoxic and cellular effects of AuNPs with two distinct morphologies (nanorods and nanostars) and two different sizes, conjugated with bioinformatically designed T-cell epitopes derived from human influenza virus. Using THP-1-derived macrophages and human primary macrophages as model APCs, we investigate how NP morphology and peptide conjugation influence cell viability, the intracellular chemical environment (in terms of pH and reactive oxygen species), and two key antigen-processing enzymatic systems associated with the MHC class I endogenous pathway (proteasome) and MHC class II exogenous pathway (cathepsin), thereby providing guidelines for the rational design of shape-optimized AuNP-based peptide nanovaccines.

## 2. Results

### 2.1. T-Cell Epitope-Based Peptide Vaccine Design

For each single epitope design (P1 and P2), a library of 10,000 randomly generated 4-mer linkers was used alongside three commonly used linkers as controls (“EAAY”, “GPGP”, and “EAAK”). This procedure yielded the final set of candidate sequences per epitope target to be scored by $S_{\mathrm{cleavage}}$ and then filtered by their physicochemical parameters. Figures S2 and S3 show the top 5% scored candidates that successfully passed the physicochemical constraints (instability index < 40 and GRAVY < 1.7) for the single-epitope constructs, detailed alongside their residue-level proteasomal cleavage profiles. The final sequence selected for the helper T-lymphocyte (HTL, MHC class II-restricted, CD4+) epitope, P1 (CALNNTWSMLVVAASIIG), utilized the optimized TWSM linker motif. This construct exhibited a low instability index of −3.62 and a GRAVY value of 1.344, confirming high structural stability and a hydrophobic profile compatible with subsequent NP conjugation (Table S2). As shown in the cleavage profile (Figure S2), the framework successfully isolated a sharp cleavage window at the linker-epitope junction (cleavage probability ≥ 0.5, highlighted in red), while maintaining near-zero cleavage probabilities throughout the core of the downstream CD4+ epitope (LVVAASIIG), safeguarding it against internal degradation.

Similarly, the candidate selected for the cytotoxic T-lymphocyte (CTL) target, P2 (CALNNTESWDPLVVAASI), leveraged an optimized TESW spacer. This sequence exhibited favorable predicted processing properties in aqueous media, with an instability index of 26.13 and a slightly hydrophilic GRAVY value of 0.533 (Table S2). The residue-level profile (Figure S3) displays a singular, highly efficient proteasomal cleavage peak (probability > 0.8) concentrated precisely at the C-terminus of the linker sequence, ensuring precise liberation of the intact CTL epitope (DPLVVAASI). The multi-epitope peptide construct P3 (CALNNTWSMLVVAASIIGRLSWDPLVVAASI) was designed following the hierarchical two-step optimization framework (Supplementary Methods S1). During the initial doublet seeding phase (epitope-linker-epitope), the framework identified RLSW as the optimal spacer between the epitopes (Figure S4), with a high-probability junctional cut capable of separating the two epitopes. In the second step, the N-terminal extension was added using the TWSM spacer to bridge the CALNN functionalization tag without disrupting the downstream processing landscape. The multiepitope sequence P3 achieved an instability index of 2.09 and a GRAVY value of 1.168 (Table S2), representing an optimal compromise between high epitope recovery potential and colloidal compatibility. The full-length processing landscape shows the presence of two high cleavage markers (red bars ≥ 0.5) matching both designed linker zones (Figure S5). Moreover, in silico antigen-processing predictions indicated that all three peptides were compatible with efficient proteasomal release of CTL epitopes, whereas cathepsin-mediated processing exhibited a promiscuous and poorly specific cleavage profile, with no individual motifs exceeding the predictive threshold (≥0.5) for high-likelihood cleavage of HTL epitopes (Figure S6).

**Figure 1:**
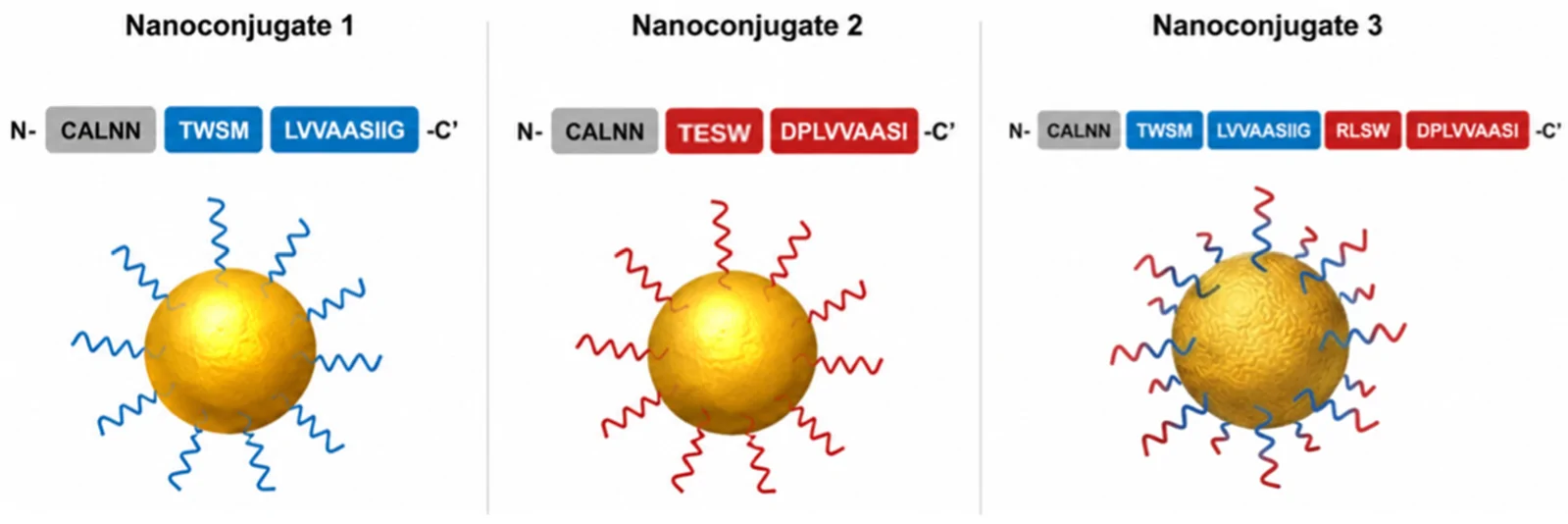
Schematic representation of the nanoconjugates developed in this study, highlighting the surface-conjugated T-cell epitope peptides and their corresponding cleavage-optimized linker sequences. The nanoconjugates contain newly designed peptide linkers (TWSM and RLSW), an HTL (MHC class II-restricted, CD4+) epitope (LVVAASIIG), and a CTL (MHC class I-restricted, CD8+) epitope (DPLVVAASI), which were predicted to efficiently target the HLA genetic background of the Chilean population.

Figure 1 illustrates the nanoconjugates developed in this study, highlighting the overall peptide architecture, including the AuNP anchoring domain (CALNN), the bioinformatically selected T-cell epitopes, and the cleavage-optimized spacer sequences designed to enhance intracellular antigen processing and MHC presentation.

### 2.2. AuNP Characterization

**Figure 2:**
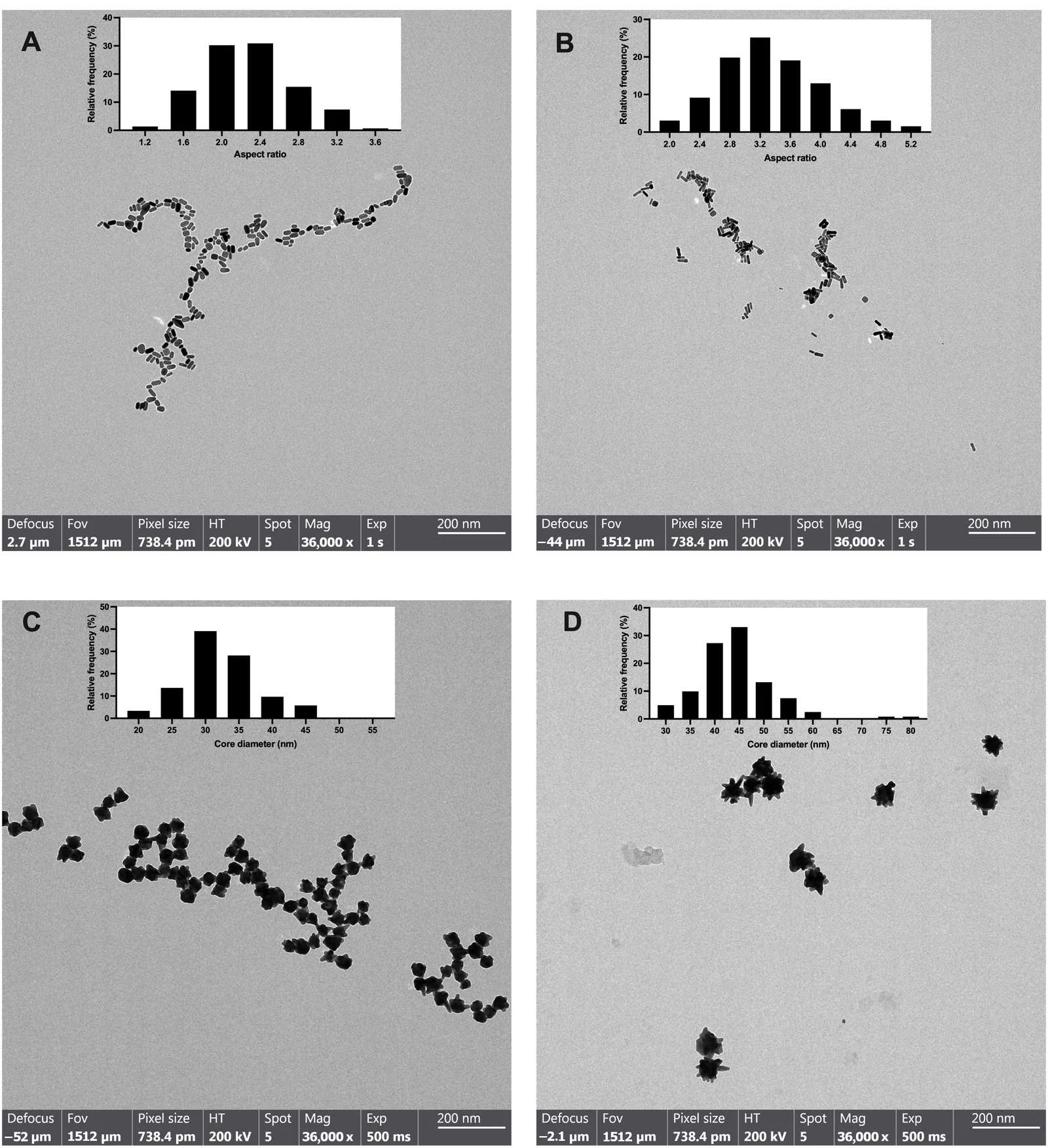
TEM micrographs and size histogram (inset graph) of NPs with different morphologies: short AuNRs prepared with 2 mM AgNO_3_ ((**A**); aspect ratio 2.3 ± 0.5), long AuNRs prepared using 10 mM AgNO_3_ ((**B**); aspect ratio 3.3 ± 0.7), small AuNSs prepared with 20 μL of seeds (**C**), and large AuNSs prepared with 5 μL (**D**). The difference in elongation between (**A**) and (**B**) is quantified in Table 1 and is independently corroborated by the red-shift in the longitudinal plasmon band from 607 to 726 nm (Figure S7).

Two anisotropic gold nanostructures, nanorods (AuNRs) and nanostars (AuNSs), were successfully synthesized in two size/shape variants each, and their morphology and monodispersity were confirmed by TEM (Figure 2).

For AuNRs, increasing the AgNO_3_ concentration from 2 to 10 mM elongated the particles, yielding short rods of 21.0 ± 4.6 × 9.2 ± 2.4 nm (Figure 2A) and long rods of 23.5 ± 4.8 × 7.2 ± 1.4 nm (Figure 2B), with corresponding aspect ratios of 2.3 ± 0.5 and 3.3 ± 0.7 (Table 1). An unpaired two-tailed *t*-test performed on the individual particle measurements confirmed that the two aspect-ratio distributions were significantly different (*p* < 0.0001). This ~43% increase in mean aspect ratio was accompanied by a red shift in the longitudinal surface plasmon resonance maximum from 607 to 726 nm, providing independent optical evidence of increased anisotropy (Figure S7).

The branched AuNSs were obtained with core diameters of 32.4 ± 5.5 nm (small, 20 µL of seeds; Figure 2C) and 44.2 ± 7.6 nm (large, 5 µL of seeds; Figure 2D), displaying a single SPR band that red-shifted from 521 to 574 nm with increasing size (Figure S8). Quantitative TEM analysis, followed by an unpaired two-tailed *t*-test of the individual particle measurements, revealed that the mean size distributions of small and large AuNSs were significantly different (*p* < 0.0001).

Consistent with their distinct surface chemistries, AuNRs carried a strong positive zeta potential (+39.29 and +31.60 mV), attributable to the CTAB bilayer, whereas the surfactant-free AuNSs were negatively charged (−25.95 and −21.13 mV). Particle concentrations determined by NTA ranged from 2.18 to 1.14 nM for rods and 0.41 to 0.35 nM for stars (Table 1).

Because the four nanostructures differ in geometry, an identical particle number concentration does not correspond to an identical mass or surface-area dose. To make the exposure metrics explicit, the geometric surface area, particle volume, gold content, and theoretical peptide-anchoring capacity of each nanostructure were computed from the TEM dimensions reported in Table 1, modeling AuNRs as spherocylinders and the AuNS cores as spheres (Table 2). All biological assays reported below were performed at a fixed particle number concentration of 10 pM (6.0 × 10^9^ particles mL^−1^), selected on the basis of the dose–response characterization previously carried out for our spherical platform in the same cell model [22]. At this concentration, AuNSs deliver 21-fold (small) to 53-fold (large) more gold mass and 6-fold to 12-fold more total geometric surface area than long AuNRs. The values reported for AuNSs are conservative lower bounds, since the spherical approximation of the core neglects the additional area contributed by the branched tips. Assuming a dense CALNN self-assembled monolayer of approximately 3 molecules nm^−2^, the theoretical anchoring capacity ranges from ~1600–1800 peptide molecules per rod to ~9900–18,400 per star; the 1:100,000 AuNP:peptide input ratio employed here corresponds to a 5-fold (large AuNSs) to 63-fold (long AuNRs) molar excess over the theoretical monolayer and should be understood as a saturating conjugation condition rather than as a measured peptide loading.

### 2.3. Cell Viability and Cytotoxicity Assays

**Figure 3:**
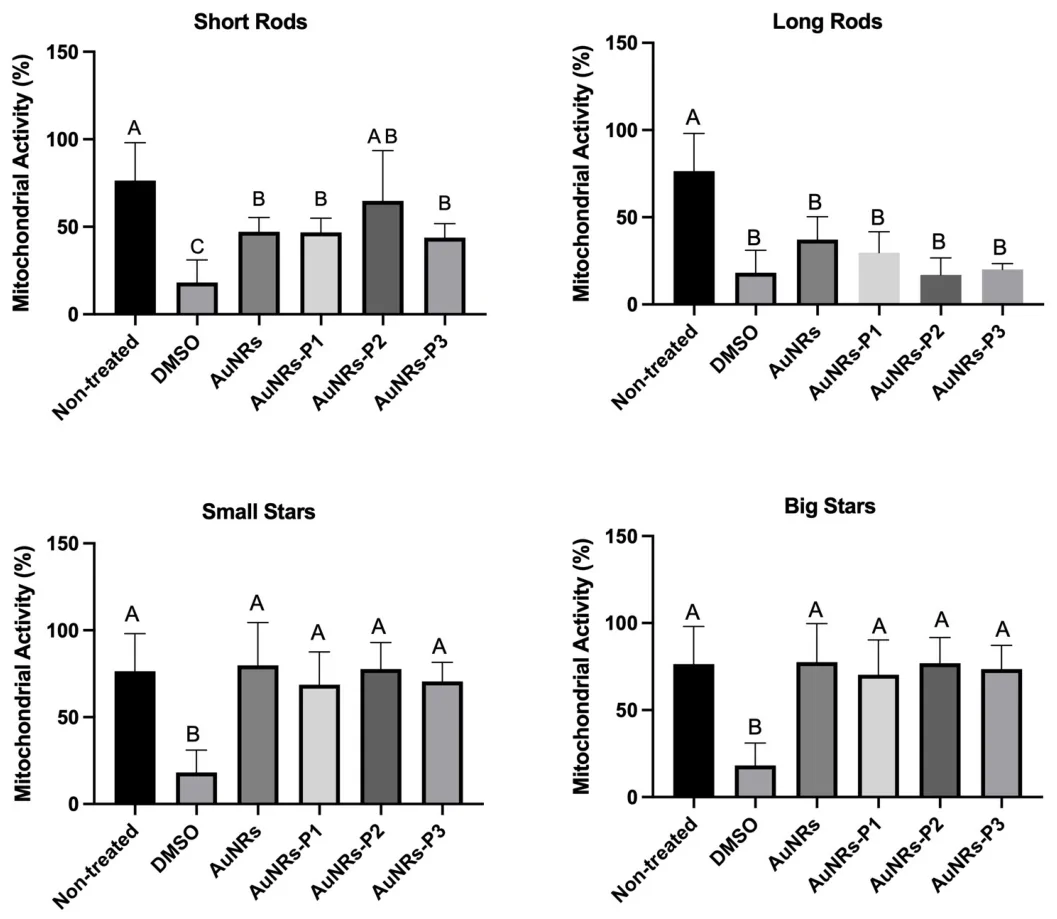
Mitochondrial activity (%) in THP-1 cells after 24 h of treatment with DMSO, AuNPs, AuNPs-P1, AuNPs-P2, or AuNPs-P3, as evaluated by MTT staining (N = 3). Uppercase letters explanation: Groups not sharing a common letter are considered statistically significantly different (*p* < 0.05), while groups sharing at least one letter do not differ significantly.

The MTT assays revealed marked morphology-dependent effects on mitochondrial activity in THP-1 macrophages exposed for 24 h to a fixed particle number concentration of 10 pM (Figure 3). For AuNRs, both short and long rods significantly reduced mitochondrial activity relative to untreated controls. Long rods produced the strongest reduction, with mitochondrial activity remaining close to 20–35% regardless of peptide functionalization. Short rods showed a slightly less pronounced effect, with AuNRs-P2 displaying the highest activity among AuNRs. By contrast, AuNSs exhibited substantially improved biocompatibility. Both small and big stars maintained mitochondrial activity near control levels, and no significant differences were detected between peptide-functionalized and non-functionalized nanostars. Quantitatively, the rank order of metabolic suppression (long AuNRs > short AuNRs ≫ AuNSs) was preserved across all three peptide constructs, underscoring that NP geometry, rather than the surface-displayed epitope, was the primary driver of the response. The near-complete preservation of mitochondrial activity by both AuNS sizes, together with the absence of significant differences between functionalized and bare AuNSs, indicates that the anisotropic star geometry is intrinsically well tolerated by THP-1 macrophages under the assayed conditions. Importantly, this ranking was obtained despite the fact that, at the equal particle number concentration employed, the AuNSs delivered a substantially higher gold mass and geometric surface-area dose than the AuNRs (Table 2). The metabolic suppression induced by AuNRs therefore cannot be attributed to a higher administered dose when normalized by either mass or geometric surface area.

**Figure 4:**
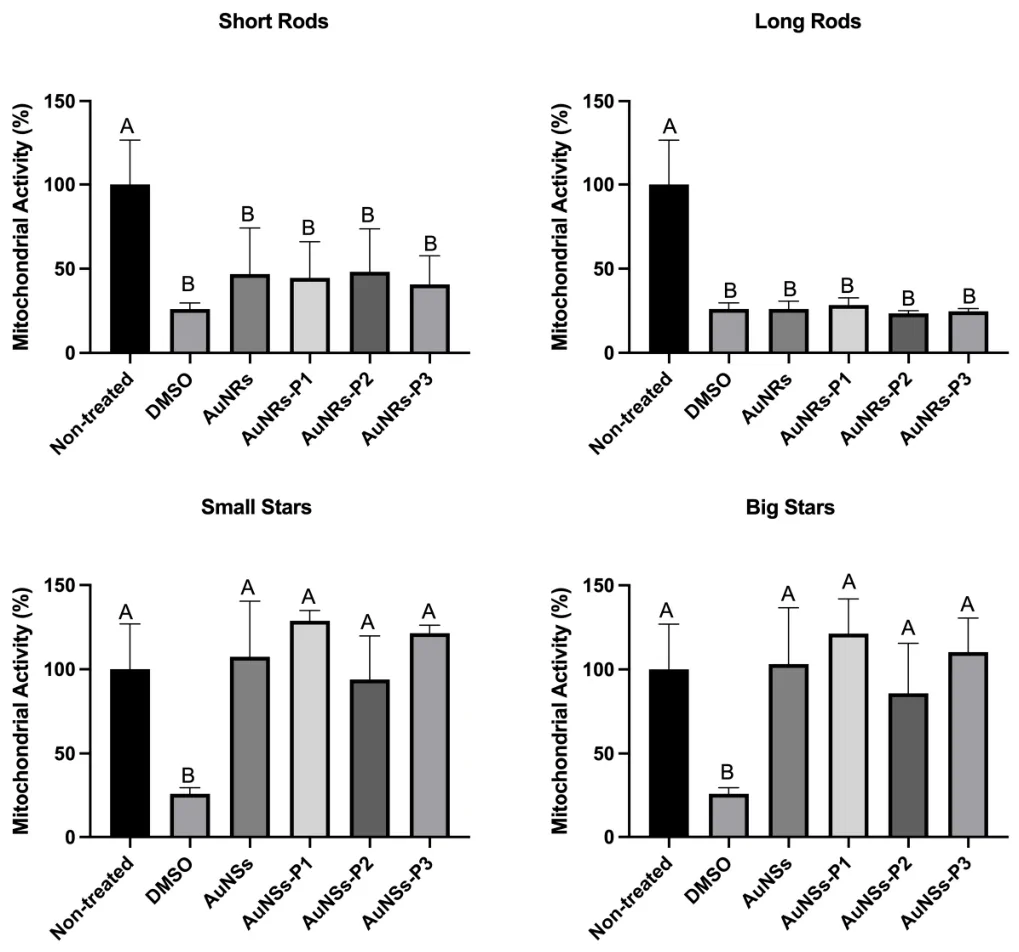
Mitochondrial activity (%) in HPMs after 24 h of treatment with DMSO, AuNPs, AuNPs-P1, AuNPs-P2, or AuNPs-P3, as evaluated by MTT staining (N = 3). Uppercase letters explanation: Groups not sharing a common letter are considered statistically significantly different (*p* < 0.05), while groups sharing at least one letter do not differ significantly.

HPMs exhibited greater sensitivity to NP morphology than THP-1 cells (Figure 4). Both short and long rods induced substantial reductions in mitochondrial activity, with long rods producing the lowest values among all nanoconjugates. Conversely, small and big stars maintained mitochondrial activity at levels comparable to untreated controls. Interestingly, AuNSs-P1 and AuNSs-P3 showed mitochondrial activity values exceeding 100%, suggesting possible metabolic activation of macrophages rather than cytotoxicity. These findings indicate that AuNSs are highly compatible with HPMs, whereas rod-shaped particles impair mitochondrial function independently of peptide conjugation.

The mitochondrial activity values exceeding 100% recorded for AuNSs-P1 and AuNSs-P3 are unlikely to reflect proliferation in these terminally differentiated, GM-CSF/LPS-polarized macrophages and more plausibly denote an increase in mitochondrial dehydrogenase activity associated with metabolic activation. This interpretation is consistent with the proteasome and cathepsin data reported below, which together point to a functional rather than cytotoxic engagement of primary macrophages by nanostar conjugates. By contrast, the pronounced rod-induced suppression, most marked for long AuNRs, paralleled the THP-1 results, indicating that rod-driven metabolic impairment is a robust, model-independent phenomenon.

**Figure 5:**
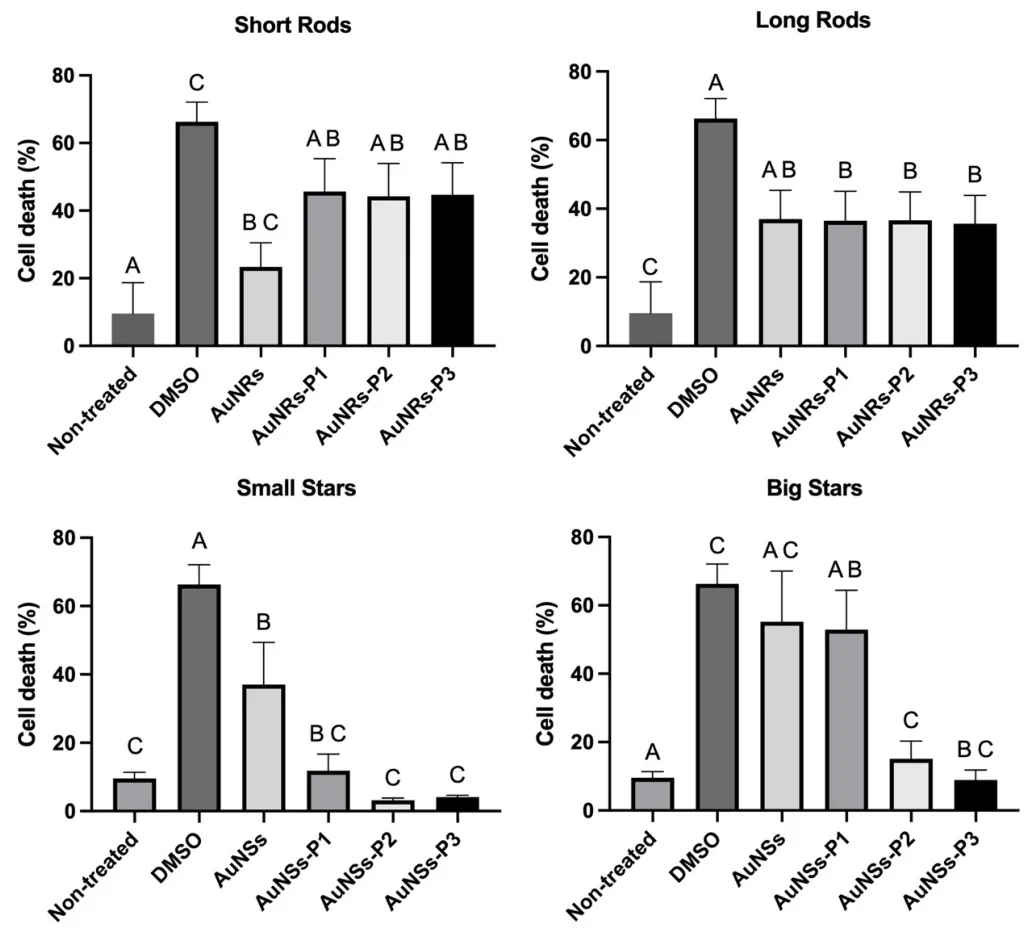
Cell death in THP-1 cells after 24 h of treatment with DMSO, AuNPs, AuNPs-P1, AuNPs-P2, or AuNPs-P3, as evaluated by Sytox staining (N = 3). Uppercase letters explanation: Groups not sharing a common letter are considered statistically significantly different (*p* < 0.05), while groups sharing at least one letter do not differ significantly.

SYTOX staining assays confirmed the trends observed in MTT assays (Figure 5). Among AuNRs, unconjugated AuNRs induced moderate cell death, whereas peptide-functionalized rods generally increased cell death levels, particularly P1-functionalized nanoconjugates. A contrasting pattern was observed for AuNSs. Small stars functionalized with P2 or P3 reduced cell death to values close to untreated controls. Similarly, big stars conjugated with P2 and P3 exhibited markedly lower cytotoxicity than unconjugated AuNSs. Importantly, the divergent effect of peptide conjugation on the two morphologies was reproducible: epitope display tended to aggravate the cytotoxicity of nanorods (most evidently for P1) while mitigating that of AuNSs (most evidently for P2 and P3). These results suggest that peptide functionalization improves the cytocompatibility of nanostars but not necessarily that of nanorods.

**Figure 6:**
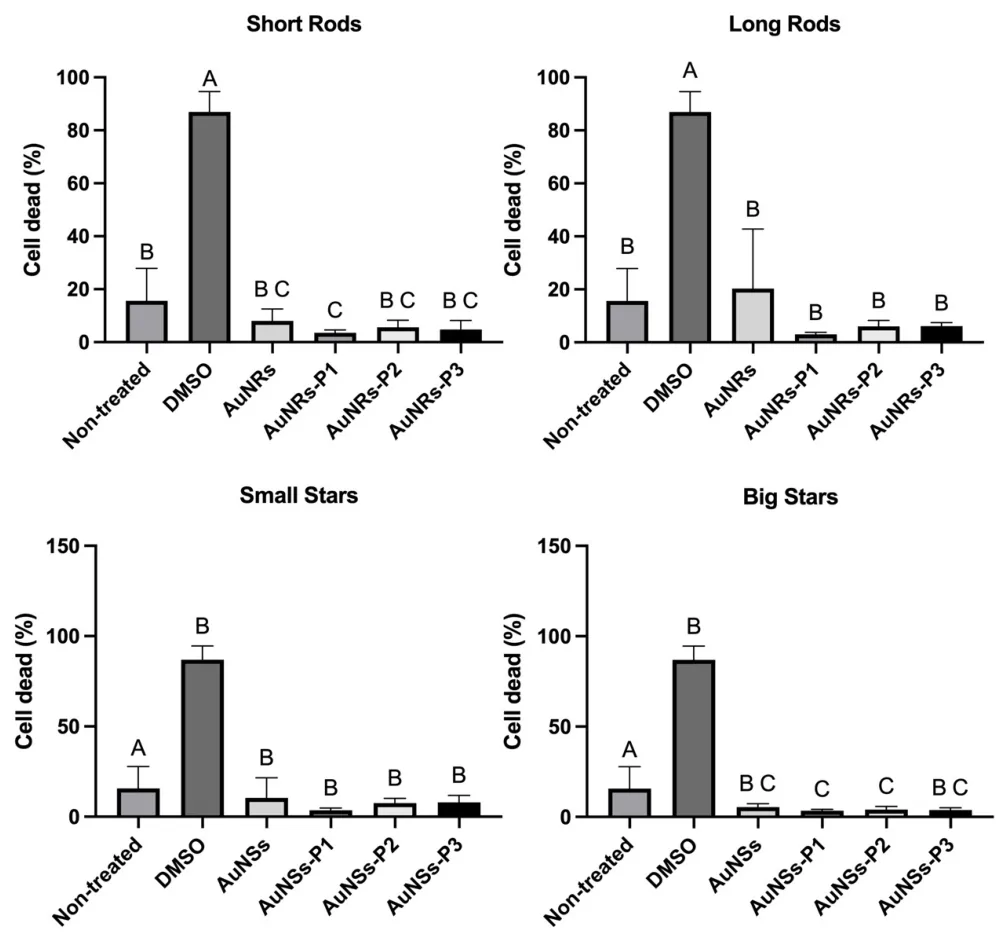
Cell death in HPM cells after 24 h of treatment with DMSO, AuNPs, AuNPs-P1, AuNPs-P2, or AuNPs-P3, as evaluated by Sytox staining (N = 3). Uppercase letters explanation: Groups not sharing a common letter are considered statistically significantly different (*p* < 0.05), while groups sharing at least one letter do not differ significantly.

HPMs showed lower susceptibility to NP-induced death than THP-1 cells (Figure 6). For AuNRs, all nanoconjugates significantly reduced cell death relative to untreated rods, with AuNRs-P1 exhibiting the lowest values. AuNSs displayed excellent biocompatibility, with cell death levels remaining close to baseline across all nanoconjugates. Notably, big-stars functionalized with P1 and P2 exhibited the lowest overall cytotoxicity among all tested nanoconjugates.

### 2.4. Intracellular Chemical Microenvironment

**Figure 7:**
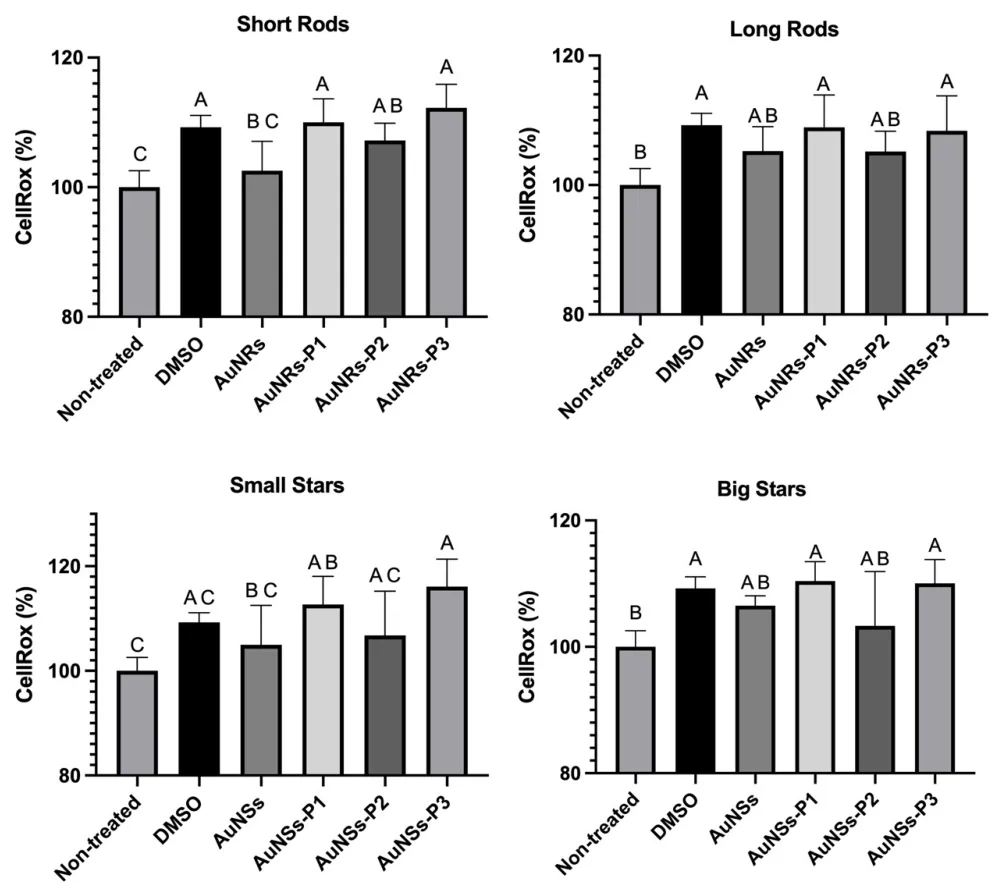
Oxidative stress in THP-1 cells after 24 h of treatment with DMSO, AuNPs, AuNPs-P1, AuNPs-P2, or AuNPs-P3, as evaluated by CellROX staining (N = 3). Uppercase letters explanation: Groups not sharing a common letter are considered statistically significantly different (*p* < 0.05), while groups sharing at least one letter do not differ significantly.

Exposure of THP-1 macrophages to the nanoconjugates induced only moderate oxidative stress (Figure 7). Rod-shaped NPs generated a modest increase in ROS production, particularly after P1 and P3 conjugation. AuNSs showed a similar trend, although ROS induction remained relatively low. Importantly, oxidative stress values remained within a narrow range (≈100–115% of control), indicating that ROS generation alone cannot fully explain the pronounced differences observed in mitochondrial activity. This discrepancy between ROS accumulation and metabolic impairment argues against a predominantly oxidative mechanism of rod toxicity and points instead toward ROS-independent processes, such as a high intracellular particle burden and the engagement of protein quality-control pathways.

**Figure 8:**
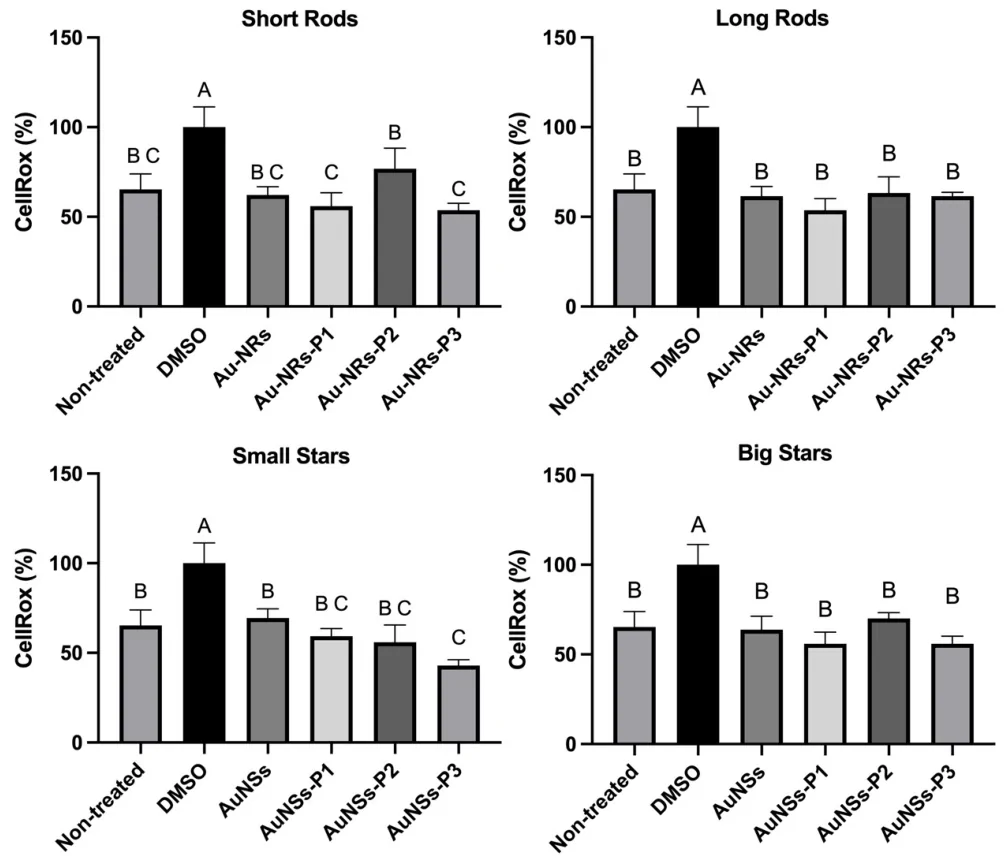
Oxidative stress in HPM cells after 24 h of treatment with DMSO, AuNPs, AuNPs-P1, AuNPs-P2, or AuNPs-P3, as evaluated by CellROX staining (N = 3). Uppercase letters explanation: Groups not sharing a common letter are considered statistically significantly different (*p* < 0.05), while groups sharing at least one letter do not differ significantly.

In contrast to THP-1 cells, HPMs exhibited reduced ROS levels following NP exposure compared with DMSO controls, with the lowest oxidative stress signals observed for AuNSs-P3 and AuNRs-P1 (Figure 8). These results suggest that NP exposure did not impose a net oxidative burden on primary macrophages under the assayed conditions. Instead, the reduced CellROX signal may reflect a cytoprotective adaptation, potentially supported by the greater antioxidant buffering capacity of primary macrophages compared with THP-1 cells.

**Figure 9:**
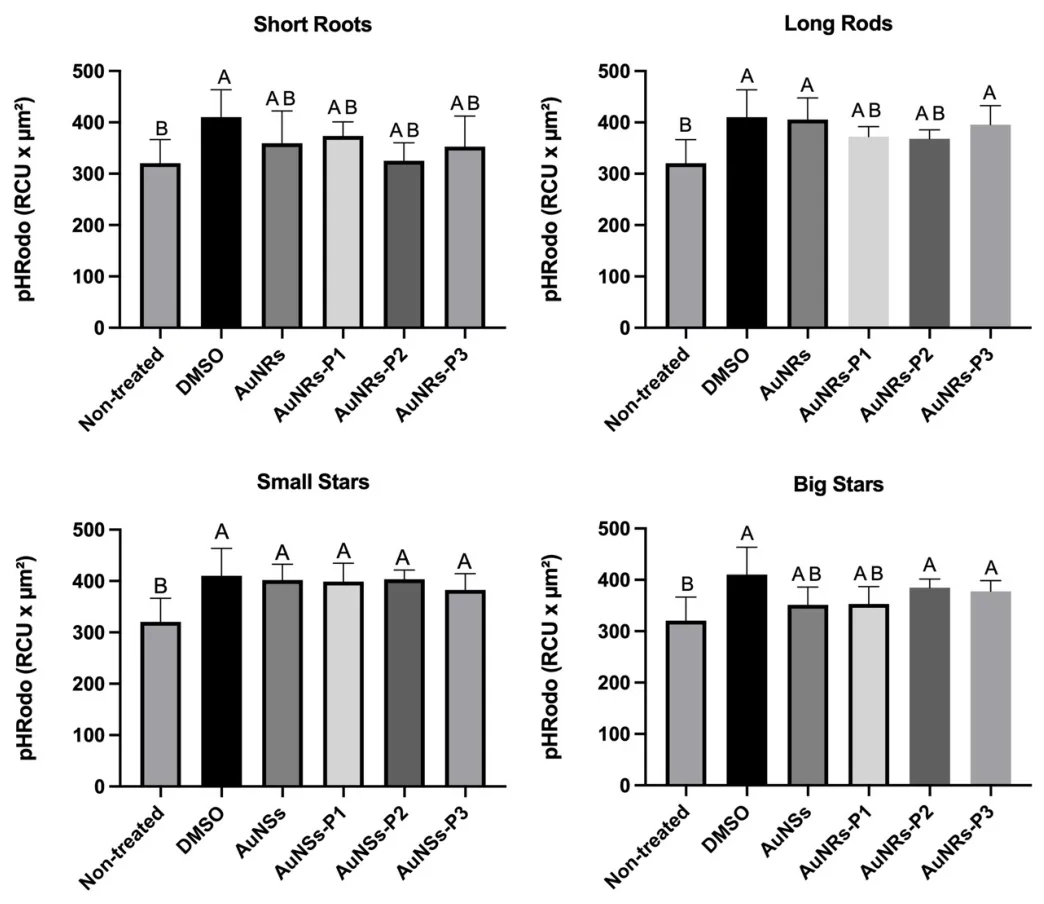
Intracellular pH of THP-1 macrophages after 20 h of treatment with DMSO, AuNPs, AuNPs-P1, AuNPs-P2, or AuNPs-P3, as evaluated by pHrodo assay (N = 3). Uppercase letters explanation: Groups not sharing a common letter are considered statistically significantly different (*p* < 0.05), while groups sharing at least one letter do not differ significantly.

THP-1 macrophages treated with all the nanoconjugates exhibited higher pHrodo-derived acidification signals than untreated cells (Figure 9). It should be noted that this probe reports the acidity of the compartment in which it resides and therefore constitutes an indirect readout. An increased signal is consistent with nanoconjugate internalization followed by trafficking to acidic endosomal and lysosomal compartments but does not, by itself, directly demonstrate particle uptake or establish the subcellular localization of the gold cores. Within this limitation, AuNSs produced strong intracellular acidification while preserving mitochondrial activity and cell viability (Figure 3 and Figure 5). Peptide functionalization did not significantly modify the acidification response, suggesting that the process was primarily governed by the physicochemical properties of the NP core rather than by the surface-displayed epitope. All the measurements reported in this section were obtained in THP-1 macrophages, while intracellular pH was not assessed in HPMs.

### 2.5. Enzymatic Activities Associated with Antigen Processing

**Figure 10:**
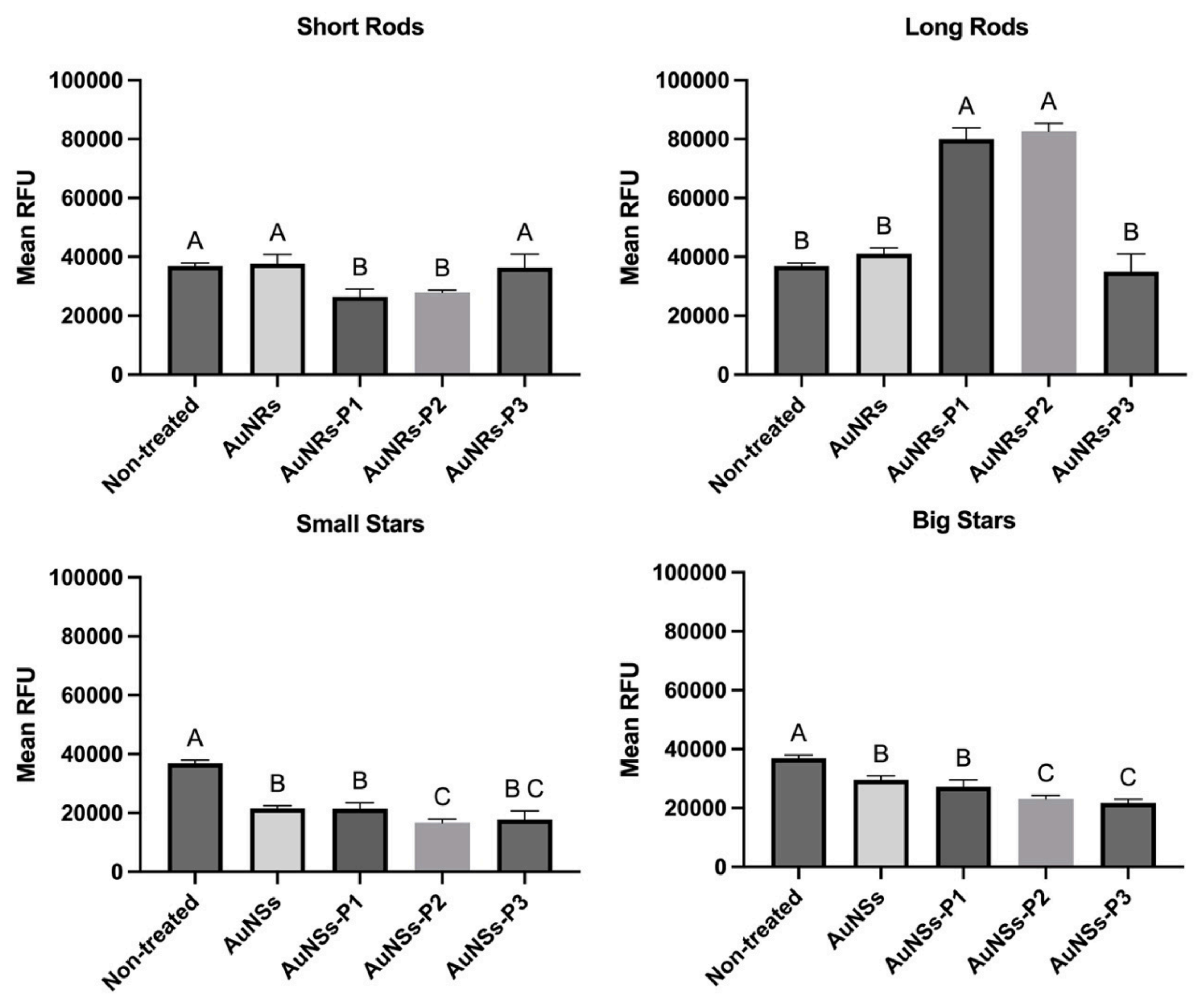
20S proteasome activity of HPM after 24 h of treatment with DMSO, AuNPs, AuNPs-P1, AuNPs-P2, or AuNPs-P3, as evaluated by the 20S proteasome activity assay (N = 3). Uppercase letters explanation: Groups not sharing a common letter are considered statistically significantly different (*p* < 0.05), while groups sharing at least one letter do not differ significantly.

20S proteasome activity was strongly influenced by NP morphology (Figure 10). Long rods induced the highest proteasomal activity, particularly AuNRs-P1 and AuNRs-P2, which produced approximately two-fold higher signals than controls. Conversely, AuNSs significantly reduced proteasome activity relative to untreated cells. These results are indicative of a morphology-dependent modulation of proteasomal capacity in HPM rather than direct evidence of epitope processing, as this assay quantifies the aggregate catalytic capacity of the cellular proteasome pool and not the degradation of the conjugated constructs themselves.

**Figure 11:**
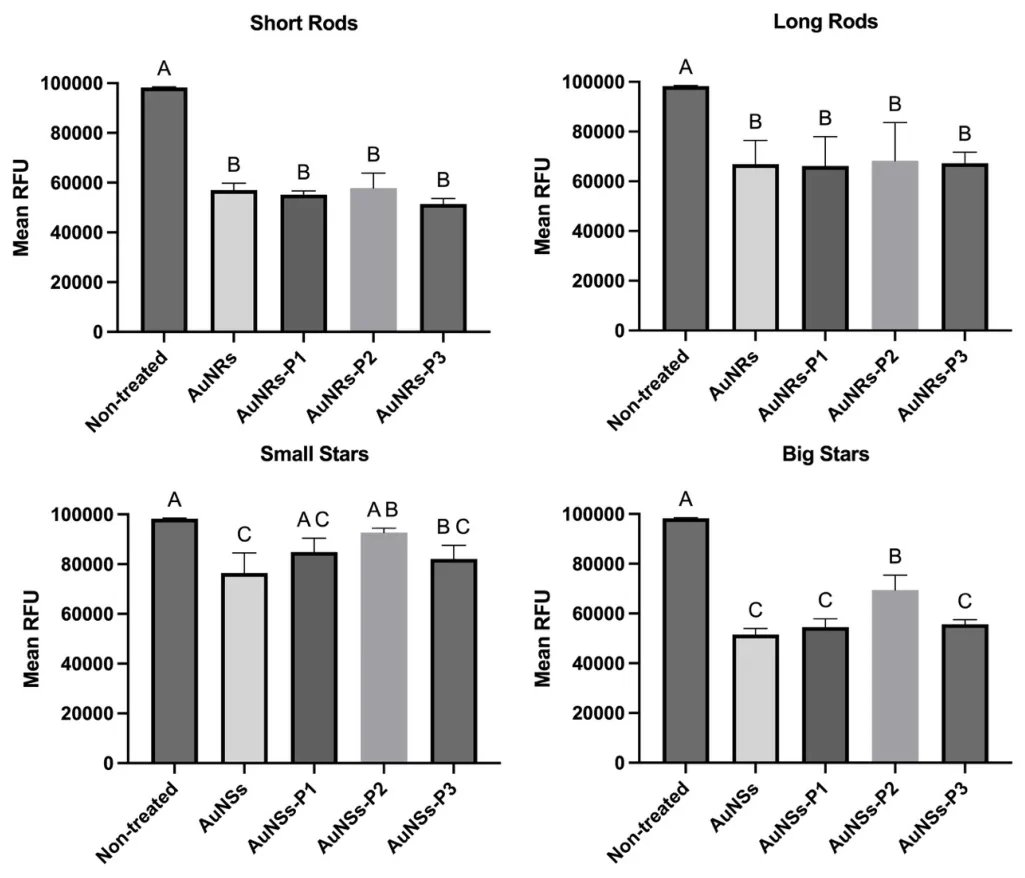
Cathepsin B activity of HPMs after 24 h of treatment with DMSO, AuNPs, AuNPs-P1, AuNPs-P2, or AuNPs-P3, as evaluated by the cathepsin activity assay (N = 3). Uppercase letters explanation: Groups not sharing a common letter are considered statistically significantly different (*p* < 0.05), while groups sharing at least one letter do not differ significantly.

Cathepsin B activity exhibited morphology-dependent modulation (Figure 11). AuNRs decreased cathepsin B activity relative to untreated controls, although values remained relatively high. However, P2 functionalization increased cathepsin B activity in AuNSs, suggesting that, in this case, the peptide sequence exerted a significant influence on the endolysosomal degradative environment of HPMs.

## 3. Discussion

Synthetic peptide vaccines offer several advantages, including high antigenic specificity, favorable safety profiles, and the possibility of excluding non-protective or potentially deleterious antigenic regions. Furthermore, while the efficacy of traditional vaccines is often associated with the induction of neutralizing antibodies, the incorporation of T-cell epitopes capable of engaging multiple arms of the immune system supports a more prominent role for cell-mediated immunity and the induction of a large range of immune specificities [23]. In addition, multi-epitope peptide vaccines offer the possibility of promoting HLA-restricted immune responses by inducing broad repertoires of T-cell specificities. A substantial body of evidence indicates that the CTL epitopes are key mediators of protection against intracellular infections [8]. Accordingly, the inclusion of the HTL epitopes in vaccine formulations is essential to provide cognate helper functions and to promote robust immune responses involving both strong CTL activation and the generation of neutralizing antibodies [9,24]. However, peptides often exhibit poor intrinsic immunogenicity, limited stability, rapid degradation, and inefficient cellular uptake, which can restrict their capacity to induce robust immune responses. NP-based delivery systems provide an attractive strategy to overcome these limitations by protecting peptide antigens, improving their cellular internalization, promoting uptake by antigen-presenting cells, and facilitating antigen processing and presentation. In this context, peptide-functionalized AuNPs may act not only as antigen carriers but also as immunomodulatory platforms capable of enhancing the immunogenic potential of peptide epitopes, thereby supporting their application in peptide-based nanovaccination strategies [25,26].

The present findings support the notion that the morphology of the NPs, together with the surface chemistry associated with each synthetic route, should be considered a primary design parameter when developing gold NP-based antigen delivery systems, with a clear distinction emerging between AuNRs and AuNSs. While peptide conjugation modulated specific cellular responses, particularly among nanostars, particle geometry consistently exerted the strongest influence on cellular behavior. This observation is consistent with a growing body of literature indicating that the physicochemical characteristics of gold nanostructures, especially size and shape, strongly influence NP-cell interactions [18,27]. Shape-dependent differences affect membrane adhesion, receptor clustering, membrane wrapping kinetics, intracellular trafficking, and eventual biological outcomes. As highlighted by Gong et al., NP geometry constitutes one of the dominant determinants of cellular internalization and intracellular fate [28]. It should be noted, however, that the literature on shape-dependent NP-cell interactions remains inconclusive. While several studies have reported that elongated, high-aspect-ratio nanorods exhibit stronger adhesion to the plasma membrane and can be efficiently internalized, others have described reduced uptake of rods compared with spherical NPs [29]. These discrepancies have been attributed, at least in part, to less favorable membrane-wrapping kinetics and to residual surfactants, particularly CTAB, which may interfere with receptor-mediated binding and cellular uptake [15,28,30]. These morphology-driven observations can be framed within our previous work on spherical peptide-functionalized AuNPs, in which NP size and surface charge emerged as the dominant determinants of macrophage cytocompatibility [22]. The present results extend this design framework from isotropic to anisotropic nanostructures: once the core dimensions are held within a comparable range, particle geometry emerges as a decisive determinant of macrophage responses, with high-aspect-ratio nanorods reproducing the pronounced mitochondrial impairment previously associated with large spheres, whereas branched nanostars preserved viability in a manner reminiscent of the biocompatible small spheres.

An important consideration when interpreting the morphology-driven trends reported here is that AuNRs and AuNSs differ not only in geometry but also in the surface chemistry inherited from their respective synthesis routes. Thus, AuNRs are stabilized by a CTAB bilayer and carry a strongly positive zeta potential (+39.29 and +31.60 mV), whereas AuNSs are surfactant-free and negatively charged (−25.95 and −21.13 mV) (Table 1). Consequently, geometry cannot be isolated from surface chemistry, and the effects described here should be understood as properties of the complete morphology-surface chemistry identity of each NP. This consideration is supported by previous studies showing that surface chemistry can strongly influence the biological responses elicited by gold nanorods. For example, Wan et al. reported that, across gold nanorods of different aspect ratios, surface chemistry rather than aspect ratio governed apoptosis, autophagy, and in vivo toxicity, and that polyelectrolyte overcoating largely abolished the toxic phenotype [31]. Similarly, Alkilany et al. showed that the cytotoxicity of CTAB-stabilized rods is dominated by surfactant that desorbs into the culture medium rather than by the particle itself, and that this effect can be suppressed by removing free CTAB or by overcoating the particles [30]. Jia et al. reached comparable conclusions in both in vitro and in vivo models and further linked residual CTAB to inflammatory activation [32], while Qiu et al. demonstrated that surface coating and aspect ratio jointly influence the cellular uptake of AuNRs [33]. Despite this covariation between geometry and surface chemistry, the present findings provide strong evidence that geometry also contributes to the observed biological divergence and that the response cannot be explained solely by surfactant content or surface charge. First, within the AuNR series, the cytotoxicity ranking was opposite to that expected from CTAB burden and cationic surface charge. Short AuNRs exhibited a larger geometric surface area per particle (607 vs. 532 nm^2^), a 1.4-fold higher gold content, and a more positive zeta potential (+39.29 vs. +31.60 mV) than long AuNRs and would therefore be expected to carry a greater CTAB-associated and cationic surface burden. Nevertheless, long AuNRs were consistently the more cytotoxic in both macrophage models (Figure 3, Figure 4, Figure 5 and Figure 6). Within this series, in which surface chemistry was comparable and core dimensions were closely matched, aspect ratio (3.3 vs. 2.3) was the parameter that most closely paralleled the observed toxicity. Second, the rod–star divergence persisted despite substantial modification of the particle interface. Chemisorption of the CALNN-anchored P1, P2, and P3 constructs reorganized the outer surface layer, yet all rod-based nanoconjugates retained a cytotoxic profile, whereas all star-based nanoconjugates remained cytocompatible. Third, exposures were normalized by particle number rather than by mass or geometric surface area. At the fixed particle concentration of 10 pM used throughout the study, AuNSs delivered approximately 21- to 53-fold more gold and 6- to 12-fold more geometric surface area than long AuNRs (Table 2). Thus, the rod-associated cytotoxicity occurred despite the rods representing the lowest administered dose among the four formulations by both metrics. Considered together, these observations point to a contribution of particle geometry to the biological response. Anisotropic NPs exhibit unique surface geometries characterized by edges, tips, and curvature gradients that can modify their interactions with cell membranes and intracellular compartments [34]. In gold nanomaterials, these effects are particularly relevant, as comparative studies have shown that nanorods, nanostars, and triangular particles display shape-dependent uptake and intracellular distribution profiles, while high-curvature or high-aspect-ratio structures may further modulate oxidative stress and macrophage-associated inflammatory responses [35]. One of the most consistent findings across both macrophage models was the marked reduction in mitochondrial activity induced by nanorods. This effect was particularly pronounced for long rods, which consistently produced the lowest MTT values in both THP-1 and primary macrophages. Interestingly, mitochondrial impairment occurred despite relatively modest increases in oxidative stress. Although ROS measurements demonstrated statistically significant differences among nanoconjugates, the levels remained within a comparatively narrow range.

Consequently, oxidative stress alone appears insufficient to explain the pronounced reductions in mitochondrial activity observed in rod-treated cells. Instead, the data suggest that AuNRs may induce a broader intracellular stress response involving disruption of organelle homeostasis and activation of protein quality-control pathways. Supporting this interpretation, proteasome activity was substantially elevated in rod-treated primary macrophages, particularly in the long rod nanoconjugates. Enhanced proteasomal activity is frequently associated with increased turnover of damaged proteins and activation of stress-response pathways. Such responses often accompany mitochondrial dysfunction and may represent an adaptive attempt to restore intracellular homeostasis. These findings align with previous reports demonstrating that anisotropic AuNPs interact differently than spherical particles with cellular membranes. Chithrani et al. showed that particle geometry strongly influences both uptake efficiency and intracellular trafficking, with elongated structures exhibiting distinct internalization kinetics compared with isotropic NPs [15,21]. Furthermore, Bartneck et al. reported rapid uptake of AuNRs by primary human phagocytes and demonstrated that nanorods can substantially influence inflammatory signaling depending on their surface chemistry [20]. A further, non-mutually exclusive contributor to rod toxicity is the macrophage-assisted extracellular dissolution of gold nanostructures with release of reactive nitrogen species, recently demonstrated for AuNPs co-incubated with THP-1-derived macrophages [36].

AuNSs were shown to preserve mitochondrial function substantially better than AuNRs, whereas peptide functionalization exerts only minor effects within each NP class. In contrast to nanorods, both small and large nanostars maintained mitochondrial activity near control levels and induced substantially lower levels of cell death. This pattern was remarkably consistent across THP-1 and primary macrophages, indicating that nanostars are intrinsically more biocompatible than nanorods within the experimental conditions employed. AuNSs deliver highly branched surfaces characterized by multiple protrusions and reduced aspect ratios compared with rods. These structural features alter NP-membrane interactions and likely influence subsequent intracellular trafficking. Importantly, the preservation of mitochondrial activity despite efficient uptake suggests that nanostars can enter THP-1 macrophages without inducing substantial metabolic stress. The observation that nanostars maintained viability while still producing detectable pHRodo signals is particularly relevant. These data indicate that AuNSs successfully reached acidic intracellular compartments but did not trigger the same degree of cellular dysfunction observed for nanorods. From a vaccination perspective, this combination of efficient uptake and minimal toxicity represents an especially desirable characteristic for delivery platforms intended for immune-cell targeting. These observations are in close agreement with Xie et al., who compared star-, rod-, and triangle-shaped AuNPs of matched size in RAW264.7 macrophages and found nanostars to be both the least internalized and entirely non-cytotoxic across a 2.5–40 µg mL^−1^ range, entering cells preferentially through clathrin-mediated endocytosis [37]. The branched multi-tipped geometry of nanostars, associated with lower effective aspect ratios and a distinct membrane wrapping behavior, may thus permit productive internalization without the membrane stress and intracellular overload imposed by rods [28,37].

Morphology was the dominant determinant of the cellular response in this study, although peptide functionalization influenced some specific biological outcomes. In particular, peptide conjugation exerted relatively limited effects on mitochondrial activity in rod-based systems. The mitochondrial activity values above 100% observed for AuNSs-P1 and AuNSs-P3 in HPMs are unlikely to reflect increased proliferation, given the terminally differentiated and GM-CSF/LPS-polarized phenotype of these macrophages (Figure 4). Instead, they likely indicate enhanced mitochondrial dehydrogenase activity associated with metabolic activation. This interpretation is consistent with the proteasome and cathepsin B data obtained in primary macrophages, which together suggest a functional, rather than cytotoxic, engagement of primary macrophages by nanostar conjugates. We note, however, that a formal demonstration of an activated phenotype would require cytokine and costimulatory-marker profiling, which was not performed here. By contrast, the pronounced suppression induced by AuNRs, particularly long rods, paralleled the response observed in THP-1 cells, indicating that rod-driven metabolic impairment is a robust and model-independent phenomenon. Furthermore, regardless of whether P1, P2, or P3 was attached, AuNRs remained substantially more toxic than nanostars (Figure 3, Figure 4 and Figure 5). By contrast, peptide conjugation had a more pronounced impact among nanostars. In several assays, P2- and P3-functionalized nanostars exhibited reduced cell death and improved cellular responses relative to unconjugated particles. These findings suggest that peptide-mediated effects become biologically relevant only after the physicochemical constraints imposed by NP morphology have been established. One possible explanation is that the peptides alter the composition of the protein corona formed around NPs following exposure to biological fluids. Changes in protein corona composition can influence receptor recognition, intracellular routing, and immune-cell activation. Because AuNSs already display favorable physicochemical properties, peptide-dependent differences may become more readily detectable than in rod-shaped systems where morphology-driven stress dominates the cellular response. This behavior contrasts with our earlier spherical system, in which the cytotoxic outcome was governed primarily by the cationic character of the conjugated peptide, with pTAT-bearing sequences acting as the main drivers of cell death rather than the NP core itself [22]. The predominance of core geometry over peptide identity observed here therefore underscores a mechanistic shift when moving from isotropic to anisotropic carriers.

The comparison between the two cellular types revealed notable differences in terms of cytotoxicity and cellular responses. Overall, HPMs tolerated the nanoconjugates markedly better than THP-1 cells, which are transformed monocytic cells characterized by altered metabolic states and reduced phenotypic complexity compared with primary macrophages. Therefore, their responses to NP exposure may exaggerate stress-related effects. The fact that similar morphology-dependent trends were observed in both models nevertheless strengthens the biological relevance of the findings and suggests that NP geometry exerts robust effects that transcend cell-specific differences. On the other hand, the formation of a peptide corona through peptide conjugation tended to reduce AuNR-associated cell death in THP-1 macrophages and partially mitigate their cytotoxicity in primary cells, whereas the intrinsic biocompatibility of AuNSs left little room for further improvement. These findings agree with previous studies questioning the interchangeability of THP-1 cells and HPMs. Tedesco et al. demonstrated that PMA-differentiated THP-1 macrophages reproduce only part of the biological response spectrum observed in blood-derived macrophages and emphasized important differences in activation, cytokine production, and functional behavior [38]. Beyond baseline phenotype, the polarization state itself shapes NP handling. For example, MacParland et al. showed that pro-inflammatory M1 stimulation (LPS/IFN-γ) reduces per-cell AuNP uptake by ~40% relative to regulatory M2 phenotypes [39], while Lee et al. reported phenotype-dependent, highly heterogeneous AuNP uptake that diverged further over time [40]. Because our THP-1 and HPM were both driven toward an M1 phenotype yet differ in origin, receptor repertoire, and metabolic state, such phenotype-linked differences provide a mechanistic basis for the lower oxidative and cytotoxic responses recorded in primary cells. Consistent with a functional engagement rather than overt toxicity, gold nanorod-based nanocomplexes have been shown to remodel the cytokine output of THP-1 macrophages, down-regulating IL-1β, IL-6, and TNF-α while up-regulating IL-10 [41]. Taken together, the concordant morphology-dependent ranking observed in both models, alongside the systematically milder responses of HPMs, indicates that THP-1 cells are suitable for rapid, standardized screening of shape-related liabilities, whereas primary macrophages are required to estimate the magnitude of biocompatibility and antigen-processing responses relevant to nanovaccine performance.

The assessment of intracellular pH in THP-1 macrophages provided valuable insight into the fate of the nanoconjugates upon internalization. All nanoconjugates generated significantly increased acidification-associated signals compared with untreated controls, which is consistent with trafficking toward acidic intracellular compartments. This observation agrees with extensive evidence showing that AuNPs are predominantly internalized through endocytic pathways and subsequently accumulate within endosomes and lysosomes. Chithrani and Chan reported that AuNPs enter cells through receptor-mediated endocytosis and then undergo intracellular trafficking through endolysosomal compartments [21]. Similarly, Bartneck et al. identified macropinocytosis as a major uptake mechanism for AuNRs in primary human phagocytes. The present pHrodo data are compatible with both rods and stars accessing acidic vesicular compartments in THP-1 macrophages. We emphasize, however, that this probe reports compartmental acidification and not particle internalization, so that these measurements provide supportive but indirect evidence and were obtained exclusively in the THP-1 model. Direct microscopic analyses of AuNPs in innate immune cells have likewise localized internalized particles within membrane-bound endolysosomal vesicles [37,42], corroborating the intracellular acidification profile observed here, and the gold-enhancement microscopy applied to our previous spherical platform in the same cell line provided direct evidence of nanoconjugate internalization by THP-1 macrophages [22]. A particle-resolved, quantitative comparison of the uptake of the four anisotropic formulations nonetheless remains to be performed.

It should also be noted that plasmonic gold nanostructures can interfere with colorimetric and fluorescence-based assays [43], and that the plasmon bands of the present formulations (Table 1) lie close to the wavelengths used for the MTT (540 nm), pHrodo Red (560/580 nm), and CellROX Deep Red (644/665 nm) readouts and for the AMC (360/480 nm) and AFC (400/505 nm) fluorogenic substrates. Because gold nanostructures act primarily as absorbers and energy acceptors within these spectral ranges, such interference would be expected to attenuate rather than to generate the signals reported here, and the proteasome and cathepsin B assays were in addition performed on washed cell lysates; formulation-specific interference controls would nonetheless be required for a complete experimental validation.

The combined proteasome and cathepsin B results indicate that the two degradative compartments were modulated in opposite directions by the two carrier types in primary human macrophages: AuNRs preferentially increased bulk proteasomal activity while attenuating cathepsin B activity, whereas AuNSs, and particularly small AuNSs-P2, enhanced lysosomal cathepsin B activity. The ~2-fold increase in chymotrypsin-like activity induced by long AuNRs-P1 and AuNRs-P2, together with the reduction observed for AuNSs, reveals a morphology-dependent divergence in the engagement of the two degradative systems. Two non-mutually exclusive interpretations are consistent with these findings. First, the increased 20S chymotrypsin-like activity may reflect an adaptive proteostatic response to intracellular AuNR accumulation and the associated proteotoxic stress, in line with the concomitant mitochondrial impairment. Second, it may indicate a shift toward immunoproteasome-associated catalytic activity, a feature linked to M1-like macrophage activation and enhanced MHC class I antigen-processing capacity [44]. It should be emphasized, however, that the assay employed here quantifies the aggregate chymotrypsin-like activity of the cellular proteasome pool in cell lysates and does not directly assess cleavage of the conjugated constructs or generation of peptide–MHC class I complexes. The present results are consistent with endosomal escape or cross-presentation, although this interpretation remains a mechanistically informed hypothesis that requires further experimental validation. Such validation could include the detection of specific peptide–MHC class I complexes using conformation-specific monoclonal antibodies [45], together with functional antigen-presentation assays, since enhanced proteolytic capacity alone is not sufficient to demonstrate cross-presentation [46].

Another question raised by these findings concerns the behavior of the multi-epitope construct. Although the in silico analysis identified high-probability cleavage sites at both junctional linkers of P3 (Figure S5), AuNRs–P3 did not increase 20S activity relative to controls, in contrast to the single-epitope constructs AuNRs–P1 and AuNRs–P2. One possible explanation relates to the greater length of P3 (31 residues versus 18 residues for P1 and P2), which may impose stronger steric constraints upon grafting to the gold surface. At the same input AuNP ratio, the longer construct may therefore achieve a lower effective surface density and adopt a more heterogeneous or sterically crowded interfacial organization. Such differences could alter the effective valency of epitope display, biomolecular corona composition, receptor engagement, and ultimately intracellular trafficking. On the other hand, cleavage probabilities are calculated for free, extended peptide sequences, whereas peptides immobilized on the AuNR surface through an N-terminal CALNN anchor are conformationally constrained and may display reduced accessibility of junctional cleavage sites to the proteasomal catalytic machinery. Thus, high in silico cleavage probability does not necessarily predict equivalent processing of the surface-bound construct in a cellular context. It should be noted, in this respect, that the absolute peptide loading per particle, conjugation efficiency, and residual free-peptide fraction were not directly quantified in the present work. Accordingly, the effective epitope density displayed by each formulation was not directly established and may vary among the different nanoconjugates.

The predicted proteolytic profiles differed markedly between cathepsin B and the proteasome. Whereas proteasomal cleavage was characterized by sharply localized, high-probability sites, cathepsin B exhibited a broader, low-amplitude cleavage profile across all three constructs (Figure S6). No individual cathepsin B cleavage site exceeded the 0.5 probability threshold, and the predicted cleavage propensity was distributed throughout the sequences, including the epitope core. This difference reflects the structure of the computational peptide-design framework, which incorporated explicit constraints for proteasomal processing but not for cathepsin B cleavage. Accordingly, the predicted endolysosomal profiles are broader across the different constructs and contain multiple low-probability cleavage sites. However, this broader pattern is consistent with the intrinsically more degenerate and proteolytically promiscuous nature of endolysosomal antigen processing compared with proteasomal processing. Although individual cathepsins exhibit distinct substrate preferences [47,48], the combined activity of endosomal and lysosomal proteases can generate heterogeneous populations of partially overlapping peptide fragments rather than a narrowly defined set of cleavage products. Consequently, peptide boundaries generated through the endolysosomal pathway are generally less strictly constrained. This redundancy is characteristic of MHC class II processing, which does not depend on a single protease. Cathepsins B and D, for example, are dispensable for class II-mediated presentation in vivo, whereas cathepsins S and L contribute more critically to antigen processing in professional antigen-presenting cells [49,50]. Thus, a broad and relatively promiscuous cleavage profile is not unexpected for a class II-directed construct and, by itself, should not be interpreted as evidence of epitope destruction. In this context, a broad cleavage landscape surrounding a promiscuous nonameric core may remain compatible with efficient presentation, provided that the relevant epitope core is preserved and remains accessible within the endolysosomal compartment [51]. This interpretation is also consistent with the functional readout obtained here, in which P2- and P3-functionalized nanostars increased, rather than suppressed, cathepsin B activity. Nevertheless, because the present measurements reflect enzymatic activity rather than direct immunological presentation, the proposed relationship between nanoparticle geometry and preferential engagement of distinct antigen-processing pathways remains a mechanistically informed hypothesis. This possibility is particularly relevant for vaccine-oriented applications, since modulation of antigen-processing and presentation routes may ultimately influence the balance between humoral and cellular immune responses [13].

Finally, our findings reveal a morphology-dependent divergence in antigen-processing-related enzymatic activity, particularly between proteasomal and cathepsin-associated pathways. A definitive characterization of macrophage polarization states, however, will require complementary profiling of cytokine secretion and costimulatory surface markers. The approximately twofold increase in 20S proteasomal activity induced by AuNRs is consistent with a functional shift that may involve enhanced immunoproteasome-associated activity, a feature commonly linked to M1-like macrophage activation. In contrast, the metabolic upregulation observed with AuNSs (>100% mitochondrial activity), together with increased cathepsin activity, suggests a non-cytotoxic functional activation characterized by enhanced endolysosomal processing. These enzymatic and metabolic signatures therefore provide a mechanistic framework for interpreting morphology-dependent macrophage responses, while their correspondence with defined polarization states remains to be established experimentally. Future immunophenotyping studies incorporating classical surface markers such as HLA-DR, CD80, and CD86, together with cytokine profiles including TNF-α, IL-6, and IL-10, will help further resolve the activation landscape and polarization phenotypes associated with these anisotropic gold nanoconjugates.

### Future Directions

The present findings establish a mechanistic framework linking gold nanocarrier morphology and peptide design with differential macrophage responses and provide several experimentally testable directions for future validation. A first priority will be to determine whether the morphology-dependent changes observed in proteasomal and lysosomal activities are translated into distinct antigen-processing and presentation outcomes. Because the current proteasome and cathepsin B assays report the catalytic capacity of two major degradative compartments rather than the generation, loading, or surface display of peptide–MHC complexes, subsequent studies should directly examine these downstream events. Detection of specific peptide–MHC class I complexes with conformation-specific monoclonal antibodies [45], quantification of surface MHC class I and HLA-DR molecules, and functional assays using epitope-specific T-cells or HLA-transgenic models will be particularly informative for testing the proposed involvement of MHC class I versus class II routing, endosomal escape, and cross-presentation. Such experiments will allow the mechanistic interpretations emerging from the present dataset to be directly connected with functional antigen-presentation outcomes. A second direction will be to establish how particle internalization contributes to the morphology-dependent cellular responses identified here. In the present study, nanoconjugate uptake was inferred indirectly through changes in endolysosomal acidification in THP-1 macrophages. Future particle-resolved analyses using ICP-MS of cell lysates, quantitative TEM, or the gold-enhancement light-microscopy approach previously applied to our spherical platform [22] could quantify the cellular uptake of each formulation and clarify whether differences in intracellular particle burden contribute to the distinct metabolic, degradative, and functional responses observed among nanostructures. Integrating these measurements with endosomal and lysosomal trafficking analyses would further help establish how nanoparticle geometry influences intracellular fate and downstream processing pathways. The robustness and biological relevance of these morphology-dependent responses should also be explored across a broader range of primary human macrophage backgrounds. Although the principal trends were reproducible among the three independent healthy donors examined here, expanding the donor cohort would allow inter-individual variability to be assessed more systematically and help determine the extent to which the observed effects are conserved across heterogeneous human populations. Finally, future studies should expand the geometric and temporal design space of the nanoconjugates. The present work focused on two AuNR populations with aspect ratios of 2.3 and 3.3 and two AuNS populations with core diameters of 32 and 44 nm, evaluated after 24 h of exposure. Extending these analyses to a broader range of aspect ratios and particle sizes, additional exposure times, and size-matched spherical controls, including those previously characterized in the same cellular model [22], would enable a more detailed mapping of the relationships between nanoparticle geometry, intracellular trafficking, degradative pathway modulation, and macrophage functional responses.

## 4. Materials and Methods

### 4.1. Reagents

Tetrachloroauric (III) acid trihydrate (HAuCl_4_ 3H_2_O; 99.9%), trisodium citrate dihydrate (Na_3_C_6_H_5_O_7_ · 2H_2_O), silver nitrate solution (AgNO_3_; 0.1 mol/L), L(+)-ascorbic acid (C_6_H_8_O_6_), 2-mercaptoethanol, HEPES, D(+)glucose anhydrous, and penicillin/streptomycin were purchased from Merck (Darmstadt, Germany). Sodium thiosulfate anhydrous (Na_2_S_2_O_3_; ≥98.0%) and hexadecyltrimethylammonium bromide (CTAB) BioXtra type for molecular biology (C_19_H_42_BrN; ≥99%) were purchased from Sigma (St. Louis, MO, USA). The Vybrant^®^ MTT Cell proliferation assay kit was purchased from Abcam (Abcam, ab211091, Cambridge, UK). SytoxGreen^TM^ was purchased from Invitrogen (Invitrogen, Carlsbad, CA, USA). pHrodo^TM^ and CellROX^TM^ were purchased from ThermoFisher (Thermo Fisher Scientific, Waltham, MA, USA). Lipopolysaccharide from E. coli 0111:B4, TLR4-based Adjuvant (LPS), was purchased from InvivoGen (Toulouse, France). RPMI 1640/L-glutamine medium and fetal bovine serum were purchased from Diagnovum (Greifswald, Germany). Phorbol-12-myristate-13-Aacetate (PMA), dimethyl sulfoxide cell culture grade (DMSO), and sodium pyruvate were purchased from Santa Cruz (Dallas, TX, USA). All the solutions were prepared with ultrapure water (18.2 MΩ) obtained from a Millipore Simplicity Water System (Merck, Milford, MA, USA).

### 4.2. In Silico Design of Immunogenic Peptide Constructs

We implemented a purpose-built computational framework to aid the design of cytosolic processing-optimized peptide constructs carrying MHC class I-restricted CD8+ T-cell epitopes (CTL epitopes) and MHC class II-restricted CD4+ T-cell epitopes (HTL epitopes) (Figure S1A). By modeling key steps of antigen processing, this strategy aims to enhance intracellular epitope generation and subsequent MHC-mediated antigen presentation. This method was integrated with the previously reported immunoinformatics tool Predivac-3.0 for population-based T-cell epitope vaccine design [52]. Briefly, we define an additive log-likelihood processing score $S_{\mathrm{cleavage}}$ that enables the identification of optimal fixed-length spacer sequences (or linkers) to maximize epitope liberation while minimizing undesired proteolytic cleavage within the epitope sequence (Figure S1B). Final single- and multi-epitope vaccine candidates were selected based on top-scoring sequences, proteasomal cleavage pattern, and two key physicochemical properties: instability index [53] and GRAVY (grand average of hydropathy) value [54]. An instability index below 40 predicts a stable peptide or protein, whereas values above 40 indicate potential instability. GRAVY values were used as an overall measure of hydrophobicity, with positive values indicating hydrophobic character and negative values indicating hydrophilicity. Briefly, sequences were retained if they ranked within the top 5% of $S_{\mathrm{cleavage}}$ scores, had a GRAVY value below 1.7, and showed an instability index below 40. Further details on the cleavage-optimized peptide design method for single- and multi-epitope constructs are provided in Supplementary Methods S1.

The matrix protein 2 (M2) of the influenza A virus (UniProt: Q76R36) was selected as a model antigen because it represents a well-established target in experimental influenza vaccine design [55,56]. Subsequent application of the bioinformatics workflow to simultaneously optimize peptide processing and theoretical coverage of the Chilean population yielded three T-cell epitope-based peptide vaccine candidates: two single-epitope constructs corresponding to the CALNN-linker-HTL epitope (CALNNTWSMLVVAASIIG) and the CALNN-linker-CTL epitope (CALNNTESWDPLVVAASI), plus one multi-epitope construct corresponding to the CALNN-linker-HTL epitope-linker-CTL epitope (CALNNTWSMLVVAASIIGRLSWDPLVVAASI).

### 4.3. Peptide Cleavage Predictions

#### 4.3.1. Putative Proteasome Cleavage Sites

The probabilities were obtained using a neural network-based model trained with a cleavage site dataset of 1.4 million sequences, presented by Dorigatti et al. [57]. Each cleavage site is captured in a window of 10 amino acids (6 to the left, 4 to the right). We use CNN and MLP architectures for the models, and pre-trained embeddings obtained from ESM-2 650M (1280 dimensions per residue) were used as input representations. Parameters and training settings were used following previous work by Ziegler et al. [58]. This model provides the probability vectors integrated into the scoring framework for candidate ranking.

#### 4.3.2. Putative Cathepsin B Cleavage Sites

The probabilities were predicted by adapting the bioinformatic algorithm described by Ferrall-Fairbanks et al. [59]. Briefly, a sliding window of eight residues scanned the sequence, scoring cleavage likelihood at each position using amino acid frequency values from a cathepsin B specificity matrix derived from the MEROPS database [57]. This matrix quantifies the propensity of residues at positions P4–P4′ to promote hydrolysis of the P1–P1′ scissile bond. To enable direct comparison across proteases, the algorithm was modified to compute a relative cleavage propensity: cumulative scores for each window were obtained by summing the individual residue scores and normalizing them against the theoretical maximum score attainable for that protease. This perfect-substrate normalization rescales the raw matrix sums onto a strict 0–1 scale, reflecting how closely a given sequence approximates the protease’s optimal substrate. For comparative visualization, the resulting 8-mer cathepsin B and 10-mer proteasomal predictions were anchored to their respective P1 residues and plotted along the annotated sequence using the Python 3.14.0 libraries DNA Features Viewer and Matplotlib 3.10.5.

### 4.4. Peptide Synthesis

The following peptides were synthesized by Shanghai Royo Biotech Co., Ltd. (Shanghai, China): p1 (CALNNTWSMLVVAASIIG); p2 (CALNNTESWDPLVVAASI); and p3 (CALNNTWSMLVVAASIIGRLSWDPLVVAASI). Peptide sequences were confirmed by mass spectrometry, and purity was determined by reverse-phase HPLC (confirming 91–93% purity).

### 4.5. Synthesis of Gold Nanorods (AuNRs)

AuNRs with average dimensions of 21.0 × 9.2 nm and 23.5 × 7.5 nm, hereafter referred to as “short AuNRs” and “long AuNRs”, respectively, were synthesized using a three-step seed-mediated growth method previously described by Adura et al. [60], with minor modifications. First, for seed synthesis, 4.7 mL of 100 mM CTAB was mixed with 42.5 μL of 29.4 mM HAuCl_4_ under stirring at 700 rpm and 28 °C for 5 min. Subsequently, 300 μL of freshly prepared, ice-cold 10 mM NaBH_4_ in 10 mM NaOH was rapidly added under the same stirring conditions and mixed for 30 s. The resulting seed solution was allowed to stand undisturbed for 20 min at 28 °C. Second, for the growth stage, 10 mL of 100 mM CTAB was mixed with 170 μL of 29.4 mM HAuCl_4_ under stirring at 700 rpm and 28 °C for 10 min. Then, 75 μL of 200 mM ascorbic acid was added and mixed for 30 s, followed by the addition of 80 μL of AgNO_3_ solution, either 2.5 mM for short AuNRs or 10 mM for long AuNRs, under the same conditions. After mixing for 30 s, 120 μL of the seed solution was added under continuous stirring, and the reaction mixtures were left undisturbed for 30 min at 28 °C to allow nanorod growth. Third, for purification, the resulting suspensions were transferred into Eppendorf tubes and centrifuged at 13,200× *g* and 28 °C for 20 min. The supernatants were discarded, and the pellets were resuspended in 1 mL of nanopure water per tube, followed by a second centrifugation step at 11,200× *g* and 28 °C for 20 min. After removal of the supernatants, the pellets were pooled and resuspended in nanopure water to a final volume of 2 mL. The purified AuNRs were stored at room temperature (RT) until further use.

### 4.6. Synthesis of Gold Nanostars (AuNSs)

The method described by De Silva et al. was used with some modifications to produce AuNSs of 32 nm (from now on referred to as “small AuNSs”) and 44 nm (from now on referred to as “big AuNSs”) [61]. All synthesis procedures were carried out at 28 °C. Briefly, 492 μL of 5.08 mM HAuCl_4_ were diluted in 10 mL of ultrapure water under stirring at 700 rpm for 1 min. Then, 20 μL of 1.0 M HCl were added, followed by the addition of variable volumes of AuNP seeds (48 nM; 20 μL for small AuNPs or 5 μL for large AuNPs). Then, 34 μL of 3 mM AgNO_3_ were added, followed 5 s later by the addition of 100 μL of 200 μM ascorbic acid, allowing the reaction to proceed for 1.5 min. The resulting AuNSs were used immediately for cellular assays or functionalization due to their low stability.

### 4.7. Optical Characterization of AuNPs

The optical properties of the AuNPs (AuNRs and AuNSs) were determined spectrophotometrically using an Epoch™ Microplate Spectrophotometer (BioTek Instruments, Winooski, VT, USA). A total of 100 μL of each AuNP solution (optical path length of 0.3 cm) was loaded in triplicate into a 96-well microplate, and absorption spectra were recorded over a wavelength range of 400–900 nm. Data acquisition and analysis were performed using Gen5 3.17 software (BioTek).

### 4.8. AuNP Concentration

The concentration of the AuNPs (for all the shapes and sizes) was determined by nanoparticle tracking analysis (NTA) using a NanoSight NS300 system (Malvern Instruments, Westborough, MA, USA), equipped with a 532 nm green laser and an sCMOS camera. Measurements were performed by recording at least three videos per sample at 22 °C, each with a duration of 60 s. Appropriate aqueous dilutions were prepared for each sample prior to analysis. Data acquisition and analysis were conducted using NTA 3.4.4 software (Malvern).

### 4.9. AuNP/Peptide Surface Coverages

The reference AuNP:peptide molar ratio employed in this study was 1:100,000, a proportion first reported by Egorova et al. and successfully applied in our previous work [22,27]. Briefly, peptides were resuspended in ultrapure water at a final concentration of 196 μM, and variable volumes (10–307 μL) were mixed with different volumes (94–997 μL) of the NP solutions, depending on the concentration of each NP preparation. Subsequently, 10% buffer (Tris-HCl 10 mM, pH 8.5) and ultrapure water were added to obtain a final volume of 1.2 mL in each case. Supplementary Methods S2 provides an example of the calculations. The mixtures were stirred at 200 rpm and 37 °C for 24 h in an orbital shaker (Labwit ZWY-103B, Shanghai, China).

### 4.10. Zeta Potential

Zeta potential measurements were performed using a zeta potentiometer (Zetasizer ProBlue, Malvern Instruments, Westborough, MA, USA) at 25 °C and a scattering angle of 90°. Data were analyzed using Malvern Zetasizer software (version 7.12). For each AuNP surface coverage condition, measurements were averaged over 30 runs.

### 4.11. Electron Microscopy

The core particle size and morphology of the NPs were determined by transmission electron microscopy (TEM) using a Talos F200C G2 microscope with 0.3 nm resolution (Thermo Fisher Scientific, Waltham, MA, USA), equipped with a Ceta 16M CMOS camera (Costa Mesa, CA, USA) and operated at an accelerating voltage of 200 kV. Size distribution histograms were generated by analyzing TEM images using ImageJ software (version 1.8.0_201, Fiji) [62].

### 4.12. Culture and Treatment of THP-1 Cells

#### 4.12.1. THP-1 Cell Culture

The human leukemia monocytic cell line THP-1 (ATCC TIB-202) was used for macrophage differentiation. THP-1 cells were cultured in RPMI 1640 medium supplemented with L-glutamine, 10% fetal bovine serum (FBS; Biological Industries, Beit-Haemek, Israel), 1% penicillin/streptomycin, 0.05 mM 2-mercaptoethanol (Merck), 1 mM sodium pyruvate (Gibco, Thermo Fisher Scientific, Waltham, MA, USA), 10 mM HEPES (Merck), and 13.9 mM glucose (Merck). Cells were maintained at 37 °C in a humidified atmosphere containing 5% CO_2_.

#### 4.12.2. THP-1 Cell Treatment

THP-1 monocytes were differentiated into M1 macrophages (hereafter referred to as THP-1 macrophages) by treatment with phorbol 12-myristate 13-acetate (PMA; 200 ng/mL) for 24 h. Subsequently, the differentiated cells were stimulated with lipopolysaccharide (LPS; 100 ng/mL) for 4 h to induce polarization toward the M1 phenotype.

### 4.13. Isolation, Culture, and Treatment of HPMs

#### 4.13.1. Blood Donor Recruitment

Healthy donors were recruited according to previously defined exclusion criteria. Prior to participation, all individuals provided written informed consent after receiving a clear explanation of the study objectives and procedures.

#### 4.13.2. Venous Blood Sample Collection by Phlebotomy

Briefly, the donor, materials, and sample collection area were prepared prior to blood collection. Clinical hand hygiene and the use of gloves were strictly observed. A total of 20 mL of whole blood was collected by phlebotomy into purple-top tubes containing K_2_-EDTA, following appropriate biosafety protocols.

#### 4.13.3. Extraction and Isolation of PBMCs by Density Gradient

Peripheral blood mononuclear cells (PBMCs) were isolated from venous blood collected by phlebotomy using Ficoll-Paque^®^ density gradient centrifugation, according to the manufacturer’s instructions (CAT 25-072-CV, Lymphocyte Separation Medium, Corning, NY, USA). Samples were centrifuged at 1500 rpm for 10 min, after which the supernatant was removed. The PBMC pellet was resuspended in 1× PBS (Cat. 46-013-CM, Corning, Corning, NY, USA), and cell counts were determined using a Neubauer chamber with 0.4% (*w*/*v*) Trypan Blue staining. Based on this count, magnetic separation of CD14+ PBMCs was performed in the subsequent step.

#### 4.13.4. PBMC-CD14+ Isolation by MACS^®^ Magnetic System

Starting from PBMCs isolated by Ficoll-Paque^®^ centrifugation, CD14+ cells were obtained using the MACS^®^ magnetic microbead system and LS column separation protocol (CAT 130-042-401, Miltenyi Biotec, Bergisch Glad-back, Germany), following the manufacturer’s instructions. Briefly, the sample was prepared prior to magnetic separation. Based on the PBMC count, the required volumes were calculated as follows: microbead buffer (80 µL per 10^7^ cells), anti-CD14 magnetic microbeads (20 µL per 10^7^ cells), wash buffer (2 mL per 10^7^ cells), and resuspension buffer (0.5 mL per 10^8^ cells). The microbead buffer consisted of 1× PBS supplemented with 0.5% BSA and 2 mM EDTA (pH 7.2), filtered through a 0.22 µm PES membrane. PBMCs were centrifuged at 1600 rpm for 12 min to pellet the cells. The pellet was resuspended in microbead buffer, and anti-CD14 microbeads (CAT 130-050-201, Miltenyi Biotec) were added, followed by gentle mixing. The suspension was incubated at 4 °C for 15 min and then washed with microbead buffer. After centrifugation at 1600 rpm for 12 min, the supernatant was discarded, and the pellet was resuspended in microbead buffer. The magnetic separation system was assembled inside a biosafety cabinet using a QuadroMACS^®^ magnetic separator, an LS MACS^®^ column, and a collection tube for the CD14-fraction. The column was primed with 3 mL of microbead buffer, ensuring it remained hydrated throughout the procedure. The prepared cell suspension was applied to the column, and the flow-through (CD14-fraction) was collected. The column was washed three times with 3 mL of microbead buffer. The column was then transferred to a new collection tube for elution of the CD14^+^ fraction. After removing the column from the magnetic field, 5 mL of microbead buffer was added, and CD14+ cells were eluted under pressure using the LS column plunger. The CD14+ cell suspension was homogenized and counted using a Neubauer chamber with 0.4% (*w*/*v*) Trypan Blue staining. Cells were then centrifuged at 1600 rpm for 12 min, and the pellet was resuspended in complete culture medium consisting of RPMI 1640 (CAT SH30255.02, Cytiva, HyClone^®^, Pittsburgh, PA, USA) supplemented with 1× penicillin/streptomycin (CAT SV30010, Cytiva, HyClone^®^) and 10% fetal bovine serum (FBS; Cytiva, HyClone^®^). Cells were plated at a density of 1 × 10^6^ cells/mL in 4 mL per 60 mm culture dish and incubated at 37 °C in a humidified atmosphere with 5% CO_2_ until the following day.

#### 4.13.5. Obtaining M0 Macrophages Ex Vivo from PBMC-CD14+ Cells

Primary CD14+ PBMCs were differentiated by exposure to recombinant human granulocyte–macrophage colony-stimulating factor (hrGM-CSF; CAT G5035-5UG, Merck) at a final concentration of 50 ng/mL in culture medium, replenished every other day for six days (2 µL hrGM-CSF per 1 mL culture medium). Day 0 (D0) was defined as the day of CD14+ PBMC isolation. On day 1 (D1), cells were cultured in medium supplemented with hrGM-CSF. On days 3 and 5 (D3 and D5), half of the culture medium was replaced with fresh medium containing hrGM-CSF. On day 7 (D7), cell adherence and morphology were evaluated. Due to their strong adherence, cells were detached using 2× trypsin (CAT 15090-046, Gibco) and subsequently counted using a Neubauer chamber with 0.4% (*w*/*v*) Trypan Blue staining. Cells were then replated in complete culture medium supplemented with hrGM-CSF under the required experimental conditions and incubated until adherence was re-established. From day 7 onward, differentiated M0 macrophages were maintained in culture medium supplemented with hrGM-CSF, with medium renewal every other day.

#### 4.13.6. Obtaining M1 Macrophages in Primary Culture

Fresh complete culture medium supplemented with lipopolysaccharide (LPS) from *Escherichia coli* O111:B4 (CAT L2630, Sigma-Aldrich, St. Louis, MO, USA) was added to a final concentration of 100 ng/mL. Cells were incubated for 4 h at 37 °C in a humidified atmosphere containing 5% CO_2_. The culture medium was then removed to eliminate the stimulus, and cells were washed twice with sterile 1× PBS. The cell monolayer was subsequently resuspended in fresh complete medium. From this point onward, ex vivo primary M1 macrophages were used for downstream experimental conditions as required.

### 4.14. Cell Viability and Cytotoxicity Assays

#### 4.14.1. THP-1 and HPM Cellular Metabolic Activity

THP-1 macrophages and HPMs were seeded in 96-well plates at a density of 1 × 10^5^ cells per well and incubated with the different nanoconjugates at a fixed particle number concentration of 10 pM for 24 h at 37 °C in a humidified atmosphere containing 5% CO_2_. Cell viability was assessed by measuring metabolic activity using the MTT assay, following the Vybrant^®^ MTT Cell Proliferation Assay Kit protocol (Thermo Fisher, Waltham, MA, USA). Briefly, after incubation with the nanoconjugates, THP-1 and HPM cells were treated with the MTT reagent (3-(4,5-dimethylthiazol-2-yl)-2,5-diphenyltetrazolium bromide) according to the manufacturer’s instructions. Absorbance was measured at 540 nm using a microplate reader (Synergy H1M, BioTek Instruments, Winooski, VT, USA). Metabolic activity was expressed as a percentage relative to the control (THP-1 macrophages or HPMs not exposed to AuNPs).

#### 4.14.2. THP-1 and HPM Cell Death

THP-1 macrophages and HPMs were seeded in 96-well plates at a density of 6 × 10^4^ cells per well and incubated with the different nanoconjugates at a fixed particle number concentration of 10 pM for 24 h at 37 °C in a humidified atmosphere containing 5% CO_2_. Cells were then treated with the fluorescent probe Sytox Green^TM^ (30 nM; Invitrogen) and monitored for 24 h using the IncuCyte^®^ S3 Live-Cell Analysis System (Sartorius, Göttingen, Germany). Real-time images were acquired at 1 h intervals and analyzed using IncuCyte^®^ S3 software. Cell death was quantified as the percentage of Sytox Green–positive cells relative to the total number of SPY620-DNA–positive cells in each image.

### 4.15. Detection of Reactive Oxygen Species (ROS)

THP-1 macrophages and HPMs were seeded in 96-well plates at a density of 1 × 10^5^ cells per well and incubated with the different nanoconjugates at a fixed particle number concentration of 10 pM for 24 h at 37 °C in a humidified atmosphere containing 5% CO_2_. Cells were then stained with CellROX^TM^ fluorescent probe (10 µM; Invitrogen) according to the manufacturer’s instructions. Fluorescence was measured at excitation/emission wavelengths of 644/665 nm, using a microplate reader (Synergy H1M BioTek Instruments, Winooski, VT, USA). ROS was expressed as a percentage relative to the control (THP-1 macrophages or HP). Intracellular ROS levels were expressed as a percentage relative to untreated control cells (THP-1 macrophages or HPMs not exposed to AuNPs).

### 4.16. Intracellular pH

THP-1 macrophages were seeded in 96-well plates at a density of 1 × 10^5^ cells per well and incubated with the different nanoconjugates at a fixed particle number concentration of 10 pM for 24 h at 37 °C in a humidified atmosphere containing 5% CO_2_. Cells were then stained with the pHrodo Red AM^TM^ fluorescent probe (10 µM; Invitrogen) and monitored for 4 h using excitation and emission wavelengths of 560 and 580 nm, respectively, with an IncuCyte^®^ S3 Live-Cell Analysis System (Sartorius, Göttingen, Germany). Real-time images were acquired at 1 h intervals, starting at 20 h after nanoconjugate exposure, and analyzed using IncuCyte^®^ S3 software. Intracellular vesicular acidification was quantified based on the fluorescence intensity detected in each image (RCU × μm^2^/Image).

### 4.17. 20S Proteasome Activity Assay

20S proteasome activity was determined using a 20S Proteasome Activity Assay Kit (ab65300; Abcam, Cambridge, UK), following the manufacturer’s protocol. Briefly, 10^5^ cells/well were seeded in 96-well culture plates and exposed to AuNPs (10 pM) for 20 h. After treatment, HPM cells were washed with PBS (pH 7.4), incubated with assay buffer, and centrifuged (500× *g*, 5 min) to discard the supernatant. Cells were lysed with lysis buffer for 30 min at room temperature under gentle agitation, followed by centrifugation (1000× *g*, 10 min), and the resulting supernatant was transferred to a black 96-well plate. A positive control was included, and background/inhibited activity was determined using a 20S proteasome inhibitor (1:10 dilution in assay buffer). The reaction was initiated by adding substrate solution to each well, and the plate was incubated at 37 °C for 1 h, protected from light. Fluorescence intensity was then measured at excitation/emission wavelengths of 360/480 nm using a fluorescence microplate reader (Synergy H1, BioTek, Winooski, VT, USA).

### 4.18. Cathepsin B Activity Assay

The cathepsin B activity assay kit (ab65300; Abcam, Cambridge, UK) was employed to determine cathepsin B activity, following the manufacturer’s protocol. HPMs were seeded in 96-well plates at a density of 1 × 10^5^ cells per well and incubated with the different conjugates at a fixed particle number concentration of 10 pM for 24 h at 37 °C in a 5% CO_2_ atmosphere. Briefly, HPMs were washed with 1X PBS and homogenized with 50 µL of lysis buffer. Then, whole-cell lysate was added to dark 96-well plates, together with 50 µL of reaction buffer and 1 µL of cathepsin B substrate Ac-RR-AFC (amino-4-trifluoromethyl coumarin, 10 mM). The plates were incubated at 37 °C for 2 h, protected from light, and the fluorescence of the cathepsin B-cleaved substrate was measured at 400/505 nm excitation/emission with a fluorescence microplate reader (Synergy H1, BioTek, Winooski, VT, USA).

### 4.19. Statistical Analysis

Quantitative data are presented as mean ± standard deviation (SD). Experiments using the THP-1 cell line were performed in three independent biological replicates, whereas primary macrophage experiments were conducted using cells isolated from three different healthy human donors (N = 3). In both models, each experimental condition was assayed in technical triplicate within every independent experiment or donor, and N therefore denotes the number of independent biological replicates (THP-1) or independent donors (HPMs). Normality was assessed using the Shapiro–Wilk test. For comparisons between two groups, either a parametric (Student’s *t*-test) or nonparametric test was applied, as appropriate. Differences among groups were evaluated by one-way analysis of variance (ANOVA). To simplify the visual representation of pairwise comparisons, results are displayed using a compact letter display (CLD), where each experimental group is assigned one or more letters derived from the post hoc analysis, such that groups not sharing a common letter are considered statistically significantly different (*p* < 0.05), while groups sharing at least one letter do not differ significantly. A *p*-value < 0.05 was considered statistically significant. All statistical analyses and graph generation were performed using GraphPad Prism (version 10.3.0).

## 5. Conclusions

The present findings provide side-by-side experimental evidence that the identity of the NP carrier, defined by its morphology together with the surface chemistry, rather than the identity of the conjugated epitope, is a major determinant of macrophage compatibility and of the intracellular degradative environment engaged by the peptide-functionalized gold nanoconjugates investigated. Among the architectures investigated, AuNSs exhibited the most favorable biological profile, preserving mitochondrial activity, minimizing cell death, maintaining low oxidative stress, and producing acidification signals consistent with efficient endolysosomal trafficking. This profile was consistently observed for both small and large AuNSs, suggesting that the low effective aspect ratio associated with their branched geometry may represent a favorable structural feature for peptide-based antigen delivery. By contrast, AuNRs induced more pronounced mitochondrial dysfunction and cell death, together with enhanced proteasomal activity. These effects may reflect a combination of intracellular particle burden and activation of proteostatic or stress-response mechanisms rather than direct oxidative damage alone. Notably, the AuNR-associated phenotype was observed despite these formulations delivering the lowest gold mass and geometric surface-area doses among the four nanoconjugates tested, arguing against a purely dose-driven explanation and supporting a contribution of particle geometry to the observed response. However, because AuNRs also retain a CTAB-associated cationic surface, the individual contributions of geometry and surface chemistry cannot be fully disentangled.

The differential modulation of proteasomal and cathepsin B activities indicates that NP morphology influences the preferential engagement of distinct intracellular degradative pathways in HPMs. In particular, the enhanced proteasomal activity associated with AuNRs and the stronger cathepsin B response elicited by selected AuNS formulations support a morphology-dependent divergence in intracellular processing. However, these findings are based on enzymatic readouts, and the proposed correspondence with MHC class I- versus class II-restricted antigen presentation must be regarded as mechanistically informed hypotheses that require further experimental validation. The lower oxidative stress and reduced susceptibility to NP-induced cytotoxicity observed in HPMs compared with THP-1-derived macrophages further emphasize the importance of validating nanovaccine platforms in biologically relevant primary human immune-cell models.

Overall, the present results indicate that AuNP carrier architecture, together with peptide functionalization, can be rationally engineered to modulate macrophage compatibility, intracellular trafficking, and antigen-processing-related pathways. Among the formulations evaluated, P2- and P3-functionalized AuNSs provided the most favorable balance between cytocompatibility, endolysosomal trafficking, and intracellular processing potential, supporting their further investigation as candidate platforms for epitope-based antigen delivery and nanovaccine development.

## Figures and Tables

**Table 1:** Key physicochemical parameters of the characterized nanoparticles.

NP	Type	Size by TEM (nm)	n	Aspect Ratio (L/W)	*p*-Value *	PZ (mV)	Conc (nM)	Wavelength (nm)	O.D.	Wavelength (nm)	O.D.
AuNRs	Short	21.0 ± 4.6 × 9.2 ± 2.4	150	2.3 ± 0.5	<0.0001	39.29	2.18	524	0.430	607	0.519
	Long	23.5 ± 4.8 × 7.2 ± 1.4	131	3.3 ± 0.7		31.60	1.14	516	0.319	726	0.766
AuNSs	Small	32.4 ± 5.5	330	-	<0.0001	−25.95	0.41	521	0.391	-	-
	Large	44.2 ± 7.6	121	-		−21.13	0.35	574	0.346	-	-

* The *p*-value corresponds to the statistical comparison of the different types of nanoparticles (Short vs. Long and Small vs. Large), using the Mann–Whitney U test. Values are expressed as mean ± SD.

**Table 2:** Geometry-derived exposure metrics for the four nanostructures at the working particle number concentration of 10 pM (6.0 × 10^9^ particles mL^−1^). Surface areas and volumes were calculated from the TEM dimensions reported in Table 1, using a spherocylinder model for AuNRs and a spherical core model for AuNSs, and a gold density of 19.3 g cm^−3^. Particle volumes were 1192 and 859 nm^3^ for short and long AuNRs and 17,809 and 45,213 nm^3^ for small and large AuNSs, corresponding to 7.0 × 10^4^, 5.1 × 10^4^, 1.05 × 10^6^, and 2.67 × 10^6^ gold atoms per particle, respectively. Theoretical peptide sites assume a dense CALNN monolayer of 3 molecules nm^−2^. Values for AuNSs are conservative lower bounds, since the spherical approximation of the core neglects the area contributed by the branched tips.

Formulation	Surface Area per NP (nm^2^)	Au Mass Dose at 10 pM (µg mL^−1^)	Total Surface Area at 10 pM (cm^2^ mL^−1^)	Theoretical Peptide Sites per NP
Short AuNRs (21.0 × 9.2 nm)	607	0.14	0.037	~1800
Long AuNRs (23.5 × 7.2 nm)	532	0.10	0.032	~1600
Small AuNSs (32.4 nm core)	3298	2.07	0.199	~9900
Large AuNSs (44.2 nm core)	6138	5.26	0.370	~18,400

## Data Availability

The data presented in this study are available on request from the corresponding author.

## Supplementary Materials

The following supporting information can be downloaded at: https://www.mdpi.com/article/10.3390/ijms27188400/s1.
