# Supplementary material for: Anisotropic Gold Nanoconjugates Bearing Immunoinformatically Designed T-Cell Epitope-Containing Peptides: Comparative Evaluation in THP-1 and Human Primary Macrophages
Source: Int J Mol Sci. 2026 Sep 21;27(18):8400. doi: 10.3390/ijms27188400 (PMC13607154; doi:10.3390/ijms27188400)
Supplement: Supplementary file 1 [file ijms-27-08400-s001.zip › ijms-4530257-supplementary.pdf]

Supplementary Material

Anisotropic Gold Nanoconjugates Bearing Immunoinformatically Designed T-Cell Epitope-Containing Peptides: Comparative Evaluation in THP-1 and Human Primary Macrophages

Braulio Contreras-Trigo [1], Enrique Guzmán-Gutiérrez [2,*], Eduardo Zúñiga [3], Fabián Sáez [2], Víctor Fica-León [1], , Simón Guerrero [3], Dariela Núñez [4,] Andrea Sánchez [2], Carmen Molina-Paris [5], Bostjan Kobe [6], Víctor Díaz-García [1] and Patricio Oyarzún [1,*]

Supplementary Methods S1

Cleavage optimized peptide design

The design of the single-epitope constructs was conducted in one-step process (Figure S1A). For each target, a population of 10,000 candidate linkers (k=4) was stochastically generated using a Monte Carlo sampling approach. Sequences were drawn from a uniform distribution of 19 natural amino acids, excluding cysteine to prevent unwanted disulfide bond formation. Each CALNN-linker-epitope candidate was evaluated using a non-linear proteasomal cleavage predictor trained to identify C-terminal cleavage sites. To rank these candidates, we defined an additive log-likelihood processing score (S_{cleavage}) that accounts for MHC-I pathway biological principles:

$$S_{cleavage} = \sum_{L \in \mathcal{L}} \ln(\max_{l \in L} P_l) + \sum_{E \in \mathcal{E}} \left[ln(P_{anchor,E}) + \sum_{c \in C} ln(1 - P_c) \right]$$

where $max\ (P_L)$ is the maximum predicted cleavage probability within the linker sequence (L), $P_{\text{anchor},E}$ is the predicted cleavage probability at the epitope C-terminal anchor residue, and P_c denotes the predicted cleavage probability at each position within the epitope core (C). The scoring function rewards high cleavage probabilities within the linker region to facilitate release of the downstream epitope, while accommodating N-terminal extensions that can subsequently be processed by aminopeptidases (e.g., ERAP1/2). In addition, it favors precise cleavage at the epitope C-terminus, a critical determinant of MHC class I antigen processing. Conversely, the term $ln \sum(1 - P_c)$ penalizes predicted cleavage events within the epitope sequence, thereby

selecting constructs with a greater likelihood of preserving epitope integrity and enhancing antigen presentation (Figure *S1*B).

The multi-epitope construct was assembled using a hierarchical two-step strategy (Figure S1A). Initially, a combinatorial 'doublet seeding' phase evaluated all possible epitope-linker-epitope pairings to identify the optimal inter-epitope spacer sequence. Subsequently, the highest-ranking doublet served as a structural template to optimize the N-terminal CALNN-linker-doublet transition. This sequential optimization ensures that the cleavage efficiency between epitopes is prioritized before finalizing the full-length construct architecture. For each construct, the linker sequence that maximized the S_{cleavage} value was selected, ensuring a design optimized for high epitope recovery and minimal neoepitope generation.

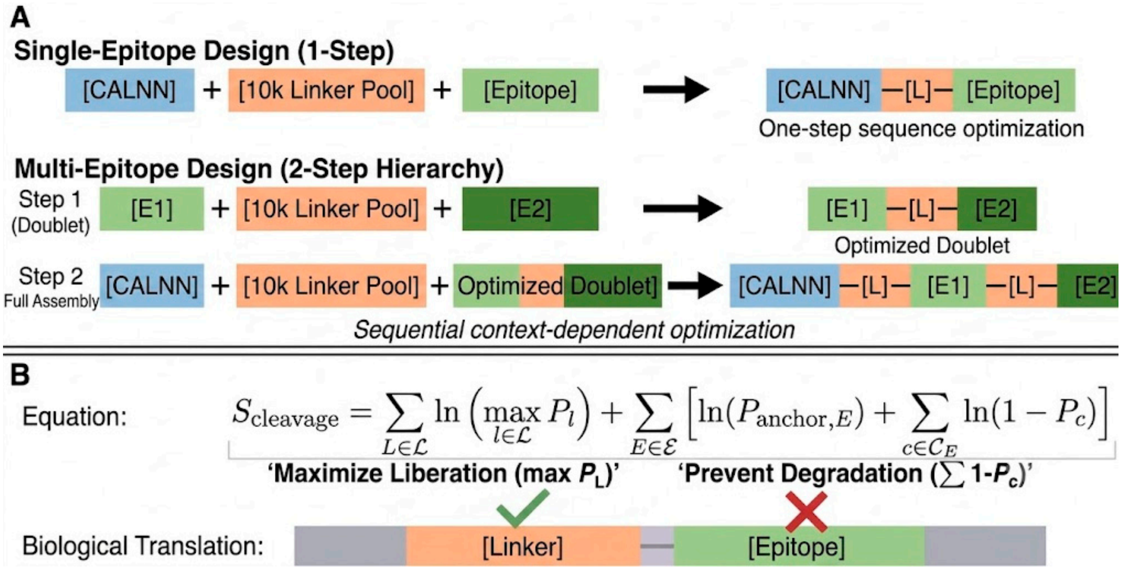

Figure S1. T-cell epitope peptide vaccine assembly and scoring method. Computational workflows for single- and multi-epitope designs. Single-epitope constructs are optimized in a single step using a 10,000-linker pool (*k*=4). Multi-epitope constructs use a two-step hierarchy:

step 1 optimizes the doublet (E_1-L-E_2), and step 2 uses this doublet as a fixed template to optimize the N-terminal CALNN attachment. (B) Mathematical formulation of the processing score (S_{cleavage}). The framework considers MHC-I processing pathway principles by maximizing cleavage within the spacer ($\max P_l$), to allow downstream epitope liberation (and subsequent N-terminal trimming), while enforcing precise cleavage at the C-terminal anchor ($P_{\text{anchor,E}}$) and minimizing internal degradation ($\sum_{c \in C} ln(1 - P_c)$), to protect the core peptide sequence.

Supplementary Methods S2

AuNP/Peptide Surface Coverages. Example of the calculations

Total volume: 1200 µL

Initial peptide concentration: 196 µM

AuNP:peptide molar ratio: 1:100.000

AuNP type	AuNP concentration (pM)	Volume AuNP for 100 pM solution (µL)	Volume peptide for 10 µM solution (µL)	Volume Tris-HCl buffer (µL)	Volume ultrapure water (µL)
AuNRs short	12,165.7	9.9	61.2	120.0	1998.9
AuNRs long	7669.9	15.6	61.2	120.0	1003.1
AuNSs small	391.0	306.9	61.2	120.0	711.8
AuNSs big	430.1	279.0	61.2	120.0	737.7

Supplementary Methods S3

Geometry-derived exposure metrics reported in Table 2. Example of the calculations

The exposure metrics reported in Table 2 of the main text were derived from the mean core dimensions measured by TEM (Table 1). AuNRs were modelled as spherocylinders, that is, a cylindrical body capped by two hemispheres, and the cores of the AuNSs were modelled as spheres. The branched tips of the nanostars were not included in the model, so the surface areas and the peptide capacities reported for the AuNSs are conservative lower bounds. The following constants and experimental parameters were used:

Density of metallic gold, $\rho = 19.3$ g cm^{-3} = 19.3×10^{-21} g nm^{-3}
Molar mass of gold, $M = 196.97$ g mol^{-1}
Avogadro constant, $N_A = 6.022 \times 10^{23}$ mol^{-1}
Working particle number concentration, $C = 10$ pM
Surface density of a dense CALNN self-assembled monolayer, $\sigma = 3$ molecules nm^{-2}

1. Particle number concentration

$$N = C \times N_A = (10 \times 10^{-12} \text{ mol L}^{-1})(6.022 \times 10^{23} \text{ mol}^{-1}) = 6.022 \times 10^{12} \text{ particles L}^{-1} = 6.0 \times 10^{9} \text{ particles mL}^{-1}$$

2. Surface area and volume of a single particle

For a spherocylinder (AuNRs) of total length L and width d, with radius $r = d/2$ and cylindrical segment $l = L - d$:

$$A = 2\pi rl + 4\pi r^2 \qquad V = \pi r^2 l + (4/3)\pi r^3$$

For a sphere (AuNS core) of diameter d and radius $r = d/2$:

$$A = 4\pi r^2 \qquad V = (4/3)\pi r^3$$

Worked example, short AuNRs (21.0 × 9.2 nm). Here $r = 9.2/2 = 4.6$ nm and $l = 21.0 - 9.2 = 11.8$ nm, so that $A = 2\pi(4.6)(11.8) + 4\pi(4.6)^2 = 341.0 + 265.9 = 607$ nm^2 and $V = \pi(4.6)^2(11.8) + (4/3)\pi(4.6)^3 = 784.5 + 407.7 = 1{,}192$ nm^3.

Worked example, small AuNSs (32.4 nm core). Here $r = 16.2$ nm, so that $A = 4\pi(16.2)^2 = 3{,}298$ nm^2 and $V = (4/3)\pi(16.2)^3 = 17{,}809$ nm^3.

3. Number of gold atoms per particle

$$n_{Au} = V \rho\, N_A / M$$

Short AuNRs: $n_{Au} = (1{,}192 \text{ nm}^3 \times 19.3 \times 10^{-21} \text{ g nm}^{-3} \times 6.022 \times 10^{23} \text{ mol}^{-1}) / 196.97 \text{ g mol}^{-1} = 7.0 \times 10^4$ atoms.

Small AuNSs: $n_{Au} = (17{,}809 \text{ nm}^3 \times 19.3 \times 10^{-21} \text{ g nm}^{-3} \times 6.022 \times 10^{23} \text{ mol}^{-1}) / 196.97 \text{ g mol}^{-1} = 1.05 \times 10^6$ atoms.

4. Gold mass dose delivered at 10 pM

$$m = N\,V\,\rho$$

Short AuNRs: $m = 6.022 \times 10^{9}$ mL^{-1} × 1,192 nm^{3} × 19.3 × 10^{-21} g nm^{-3} = 1.39 × 10^{-7} g mL^{-1} = 0.14 µg mL^{-1}.

Small AuNSs: $m = 6.022 \times 10^{9}$ mL^{-1} × 17,809 nm^{3} × 19.3 × 10^{-21} g nm^{-3} = 2.07 × 10^{-6} g mL^{-1} = 2.07 µg mL^{-1}.

5. Total geometric surface area delivered at 10 pM

$$S = N\,A,\quad \text{with } 1\ \text{nm}^2 = 10^{-14}\ \text{cm}^2$$

Short AuNRs: $S = 6.022 \times 10^{9}$ mL^{-1} × 607 nm^{2} = 3.66 × 10^{12} nm^{2} mL^{-1} = 0.037 cm^{2} mL^{-1}.

Small AuNSs: $S = 6.022 \times 10^{9}$ mL^{-1} × 3,298 nm^{2} = 1.99 × 10^{13} nm^{2} mL^{-1} = 0.199 cm^{2} mL^{-1}.

6. Theoretical peptide-anchoring capacity

$$n_{\text{pep}} = A\,\sigma$$

Short AuNRs: n_{pep} = 607 nm^{2} × 3 molecules nm^{-2} ≈ 1,800 peptide molecules per particle.

Small AuNSs: n_{pep} = 3,298 nm^{2} × 3 molecules nm^{-2} ≈ 9,900 peptide molecules per particle.

Because all conjugates were prepared at a fixed AuNP:peptide input molar ratio of 1:100,000 (Supplementary Methods 2), the corresponding molar excess over the theoretical monolayer is 100,000/n_{pep}, that is, approximately 55-fold for short AuNRs, 63-fold for long AuNRs, 10-fold for small AuNSs and 5-fold for large AuNSs. The input ratio therefore defines a saturating conjugation condition and does not represent a measured peptide loading.

Applying the same procedure to the four formulations gives the values reported in Table 2 of the main text:

Formulation	Surface area per NP (nm^{2})	Volume per NP (nm^{3})	Au atoms per NP	Au mass dose at 10 pM (µg mL^{-1})	Total surface area at 10 pM (cm^{2} mL^{-1})	Theoretical peptide sites per NP
Short AuNRs (21.0 × 9.2 nm)	607	1,192	7.0 × 10^{4}	0.14	0.037	~1,800
Long AuNRs (23.5 × 7.2 nm)	532	859	5.1 × 10^{4}	0.10	0.032	~1,600
Small AuNSs (32.4 nm core)	3,298	17,809	1.05 × 10^{6}	2.07	0.199	~9,900
Large AuNSs (44.2 nm core)	6,138	45,213	2.67 × 10^{6}	5.25	0.370	~18,400

At the fixed particle number concentration of 10 pM, the AuNSs therefore deliver approximately 21-fold (small) to 53-fold (large) more gold mass, and 6- to 12-fold more geometric surface area, than the long AuNRs. Because the spherical approximation of the AuNS core does not account for

the surface contributed by the branched tips, both the surface-area and the peptide-capacity values reported for the AuNSs are conservative lower bounds.

Supplementary Results

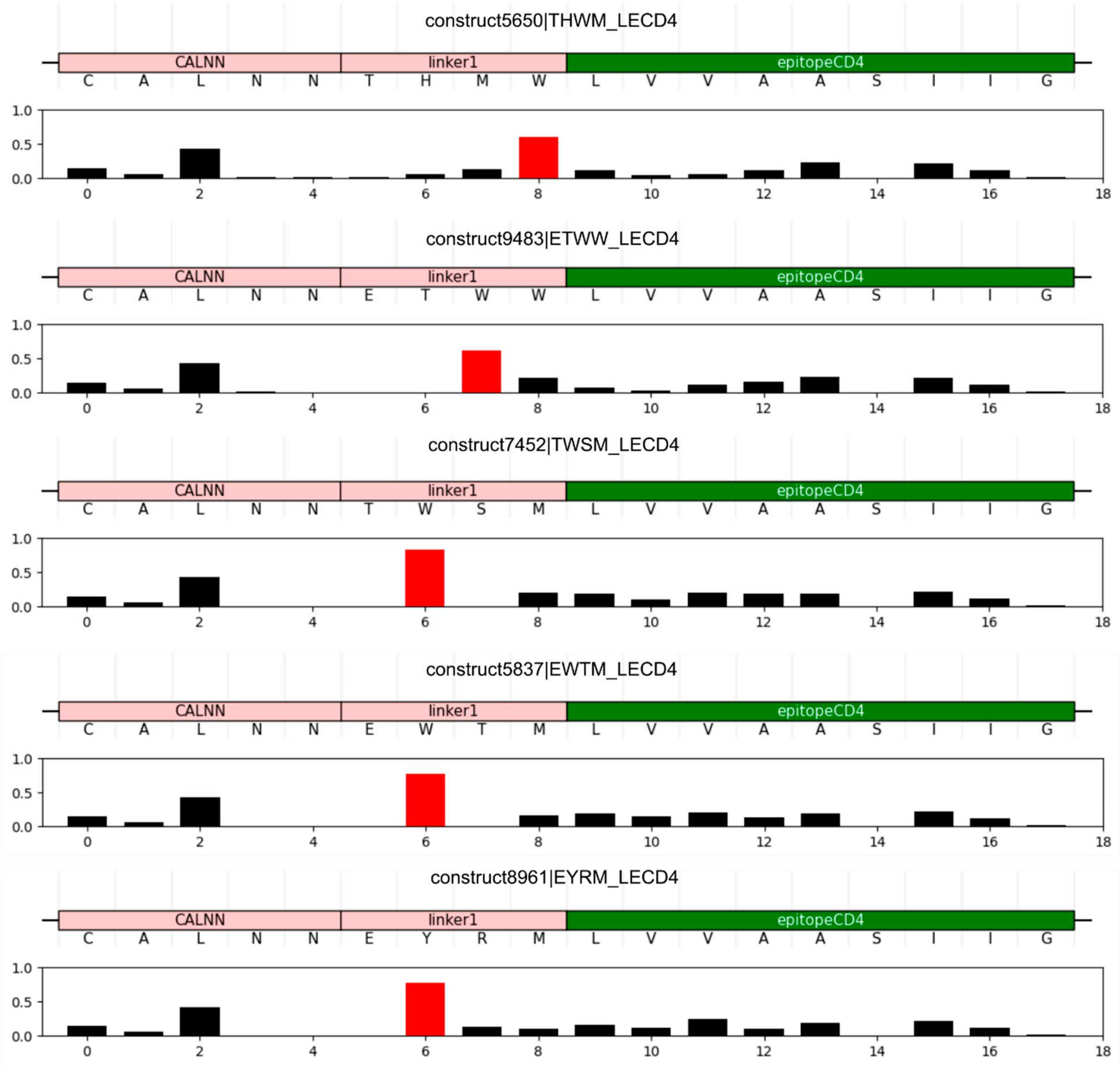

Figure S2. Scheme of top 5% selected linker-epitopeCD4 vaccine constructs and residue level cleavage probabilities. Sequences were filtered based on two key physicochemical properties: instability index and GRAVY. At the bottom of each scheme, barplot shows residue level cleavage probabilities, with red bars indicating probabilities equal or higher than 0.5.

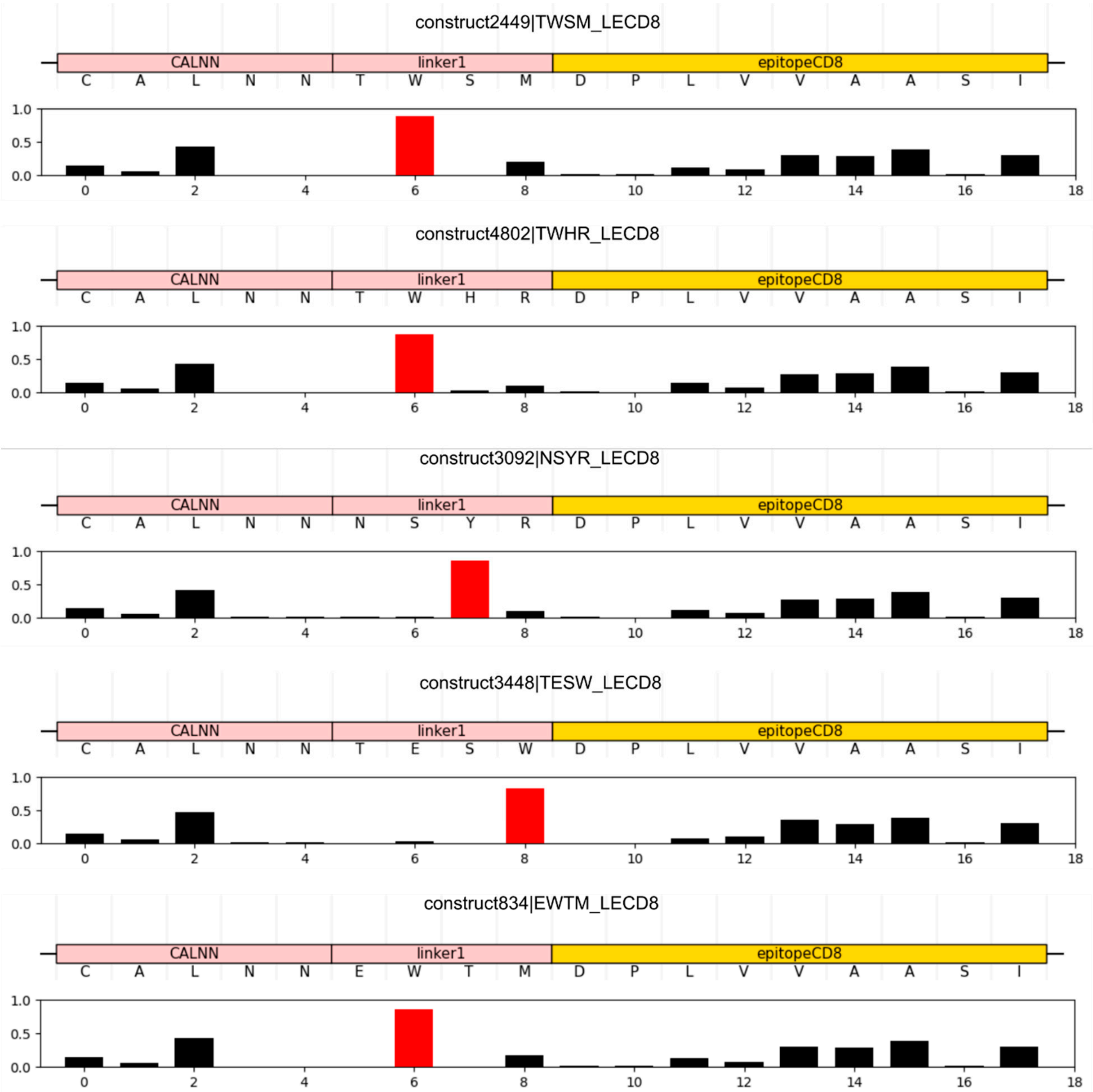

Figure S3. Scheme of top 5% selected linker-epitopeCD8 vaccine constructs and residue level cleavage probabilities. Sequences were filtered based on two key physicochemical properties: instability index and GRAVY. At the bottom of each scheme, barplot shows residue level cleavage probabilities, with red bars indicating probabilities equal or higher than 0.5.

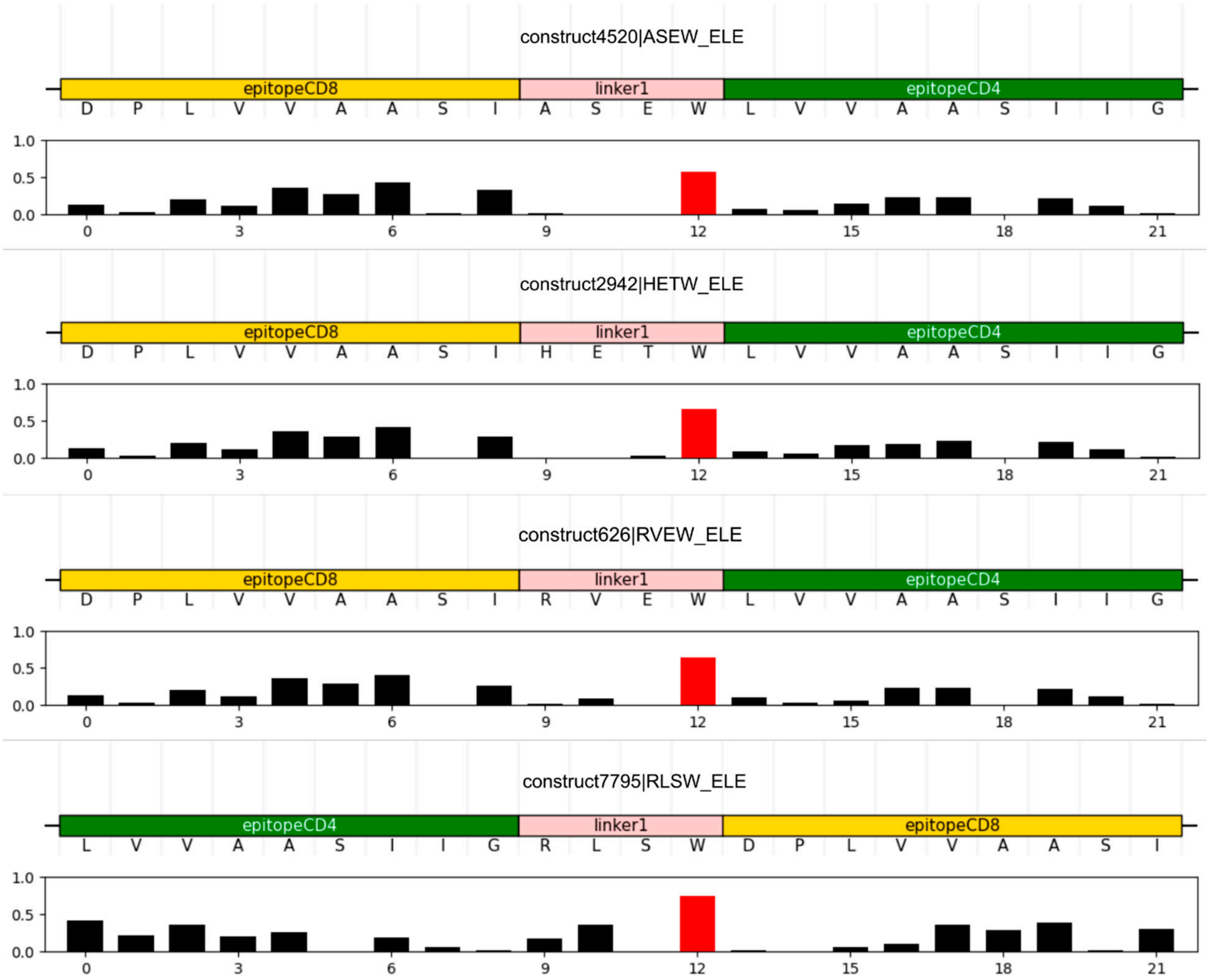

Figure S4. Scheme of top 5% selected epitope-linker-epitope vaccine constructs and residue level cleavage probabilities. Sequences were filtered based on two key physicochemical properties: instability index and GRAVY. At the bottom of each scheme, barplot shows residue level cleavage probabilities, with red bars indicating probabilities equal or higher than 0.5.

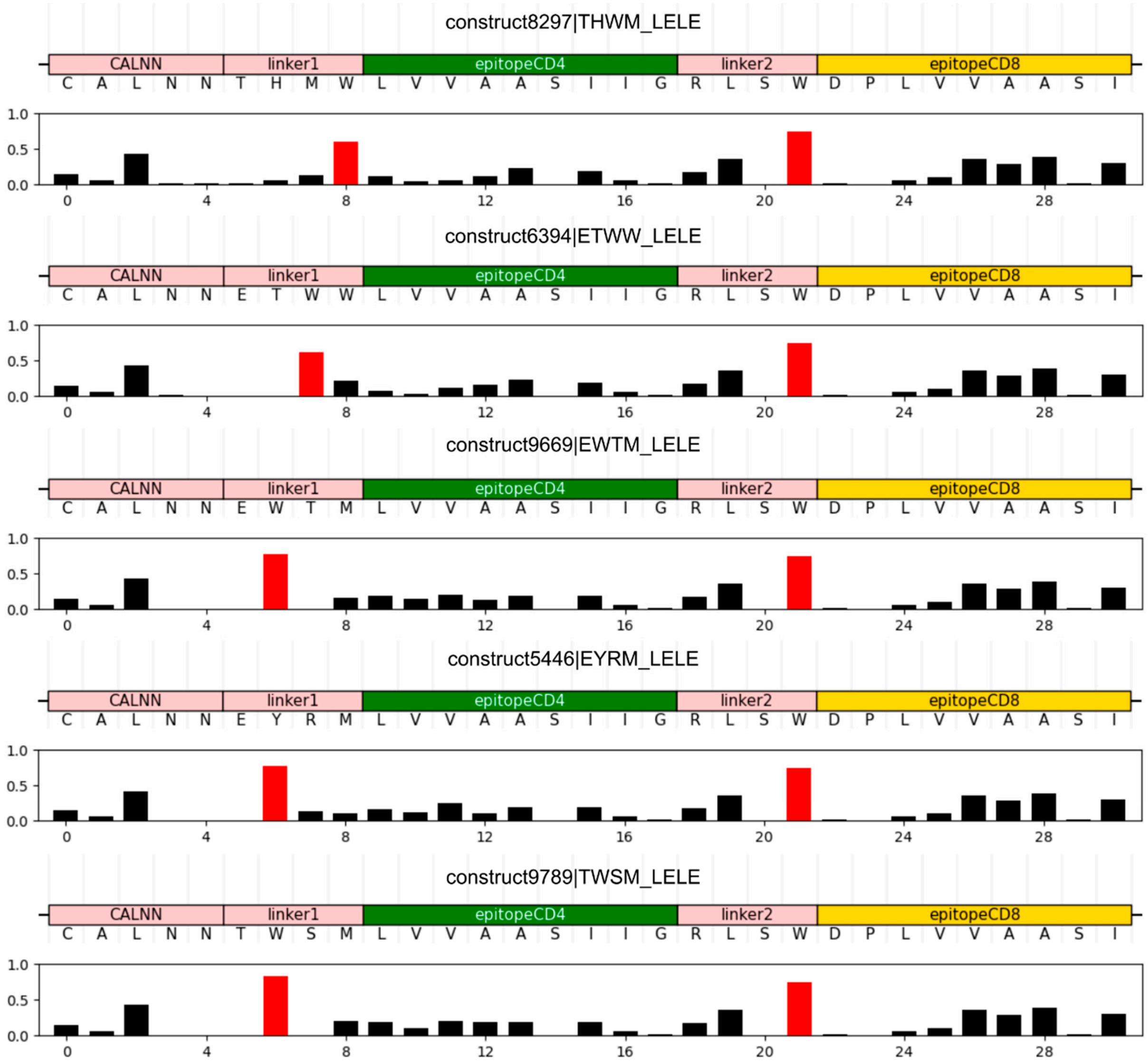

Figure S5. Scheme of top 5% selected CALNN-linker-epitope-linker-epitope vaccine constructs and residue level cleavage probabilities. Sequences were filtered based on two key physicochemical properties: instability index and GRAVY. At the bottom of each scheme, barplot shows residue level cleavage probabilities, with red bars indicating probabilities equal or higher than 0.5.

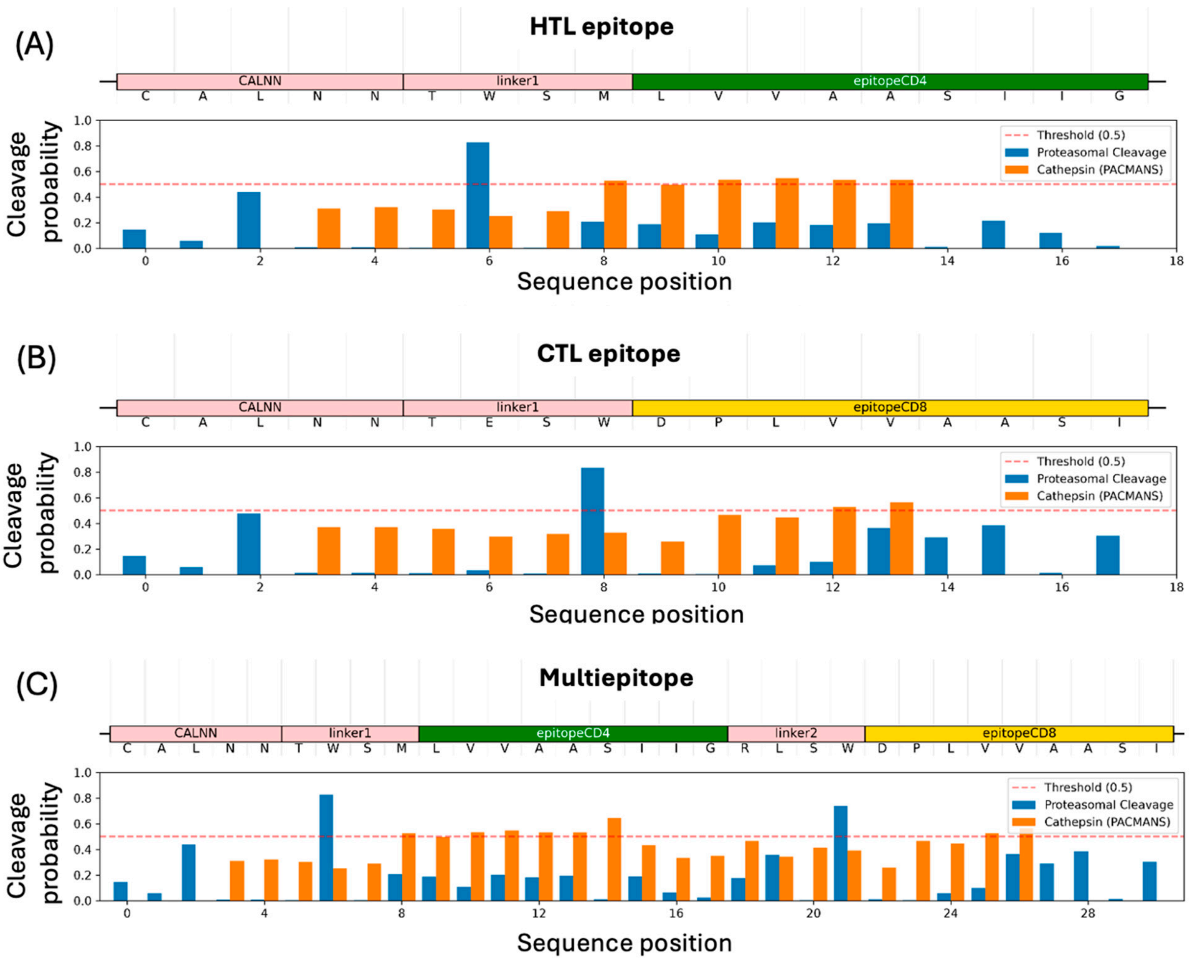

Figure S6. Predicted proteasomal and cathepsin B cleavage profiles for the peptide constructs. (A) HTL epitope peptide; (B) CTL epitope peptide; (C) multiepitope peptide. Upper panels show the schematic organization of each construct, with functional domains annotated. Lower bar charts depict residue-level cleavage probabilities mapped onto the corresponding sequence, with proteasomal cleavage probabilities (blue bars) and cathepsin B cleavage propensities (orange bars) positioned at their predicted C-terminal (P1) residues, to allow direct visualization of processing efficiency at epitope boundaries. The dashed red line indicates the 0.5 threshold above which cleavage events are considered high-likelihood.

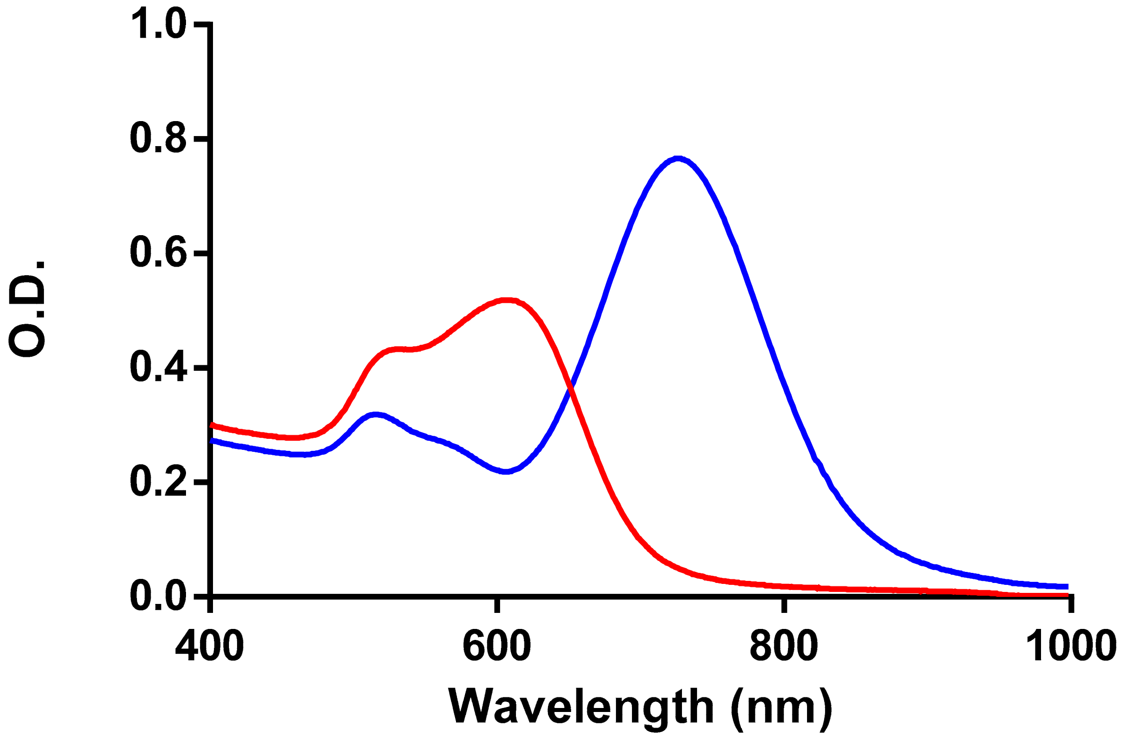

Figure S7. Visible spectrum of AuNRs. The spectra show the surface plasmon resonance (SPR) band for free AuNRs short (red line) and AuNRs long (blue line).

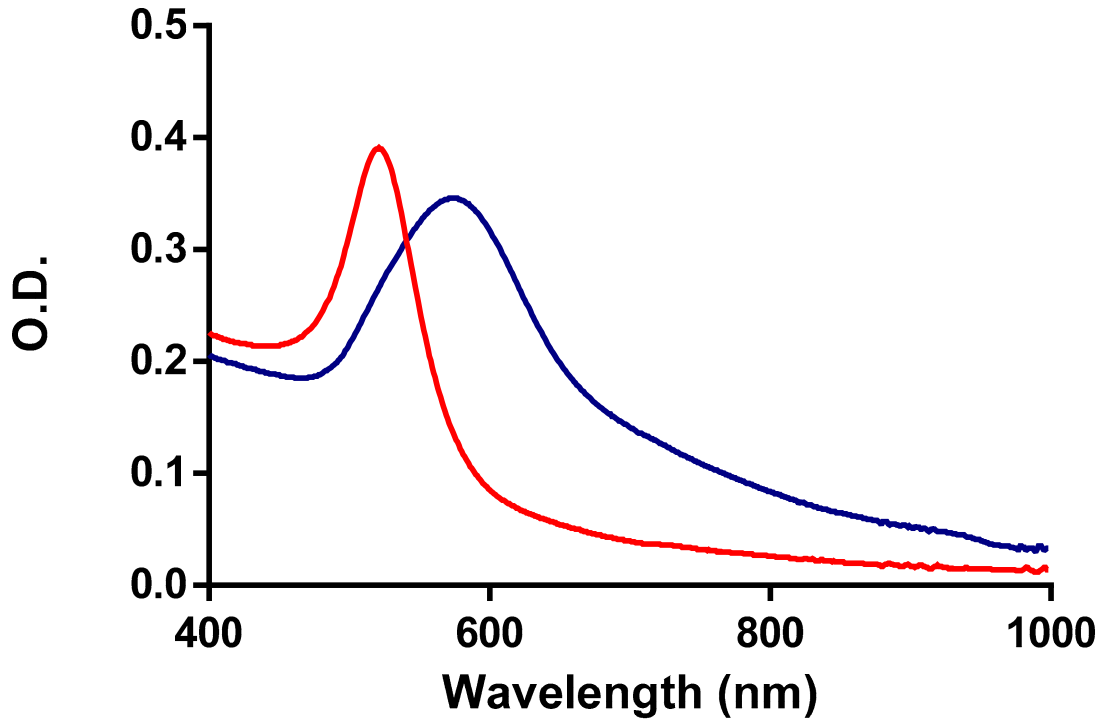

Figure S8. Visible spectrum of AuNSs. The spectra show the surface plasmon resonance (SPR) band for free AuNSs small (red line) and AuNSs large (blue line).

Table S1. Predivac-3.0 predictions (epitope discovery mode) for CD4+ and CD8+ T-cell epitopes, and population coverage for the Chilean population.

**Nonamer ID**	**Predicted T-cell epitope**	**Predicted coverage (%)**	**Alleles**	**MHC class**
1	DPLVVAASI	67.0112	HLA-B*07:02, HLA-B*07:05, HLA-B*38:01, HLA-B*39:06, HLA-B*39:10, HLA-B*39:14, HLA-B*40:01, HLA-B*40:02, HLA-B*40:10, HLA-B*55:02	I
2	LVVAASIIG	30.7443	HLA-DRB1*04:01, HLA-DRB1*04:02, HLA-DRB1*04:03, HLA-DRB1*04:04, HLA-DRB1*04:05	II

Table S2. Physicochemical properties for the top 5% scored sequences of each single and multi-epitope design.

construct	Mw (Da)	pI	Instability Index	Gravy
construct5650|THMW_LECD4	1913.27	6.73	11.58	1.211
construct9483|ETWW_LECD4	1960.26	4.05	7.95	1.039
**construct7452|TWSM_LECD4**	**1863.21**	**5.52**	**-3.62**	**1.344**
construct5837|EWTM_LECD4	1905.24	4.05	-7.26	1.194
construct8961|EYRM_LECD4	1937.29	5.99	0.05	0.961
construct2449|TWSM_LECD8	1905.20	4.05	-3.62	0.833
construct4802|TWHR_LECD8	1980.25	6.74	9.53	0.344
construct3092|NSYR_LECD8	1920.15	5.83	0.05	0.300
construct3448|TESW_LECD8	1903.12	4.05	26.13	0.533
construct834|EWTM_LECD8	1947.24	4.05	-7.26	0.683
construct4250|ASEW_ELE	2181.53	4.05	17.08	1.573
construct2942|HETW_ELE	2261.61	4.35	13.93	1.350
construct626|RVEW_ELE	2278.69	4.37	8.32	1.514
construct7795|RLSW_ELE	2250.68	5.84	9.55	1.618
construct8297|THMW_LELE	3321.91	6.74	10.92	1.090
construct6394|ETWW_LELE	3368.90	4.37	8.81	0.990
construct9789|TWSM_LELE	3271.85	5.83	2.09	1.168
construct9669|EWTM_LELE	3313.89	4.37	-0.02	1.081
construct5446|EYRM_LELE	3345.93	6.06	4.22	0.945

LECD4: Single epitope design of Linker-HTL epitope, LECD8: single epitope design of linker-CTL epitope. ELE: step 1 of multiepitope design epitope-linker-epitope. LELE: step 2 of multiepitope design CALNN-linker-epitope-linker-epitope.
